# Comparative GC–MS based nutrients profiling of less explored legume seeds of *Melilotus, Medicago, Trifolium,* and *Ononis* analysed using chemometric tools

**DOI:** 10.1038/s41598-023-45453-0

**Published:** 2023-10-25

**Authors:** Heba A. Fahmy, Sherine El-Shamy, Mohamed A. Farag

**Affiliations:** 1 Pharmacognosy Department, Faculty of Pharmacy, Modern University for Technology and Information, Cairo, Egypt; 2 Pharmacognosy Department, College of Pharmacy, Cairo University, Kasr El Aini St., P.B. 11562, Cairo, Egypt

**Keywords:** Plant sciences, Chemistry

## Abstract

Exploring novel sources of plant protein for nutrition of both humans and animals is motivated mainly by its growing demand worldwide, besides identifying healthy alternatives for animal protein. The present study evaluates metabolome diversity within 15 legume seed species. The examined samples comprised three *Melilotus*, four *Medicago*, four *Trifolium*, and four *Ononis* seed species. A holistic approach for metabolites profiling using gas chromatography-mass spectrometry (GC–MS) led to the annotation and quantification of 87 metabolites comprising alcohols, free amino acids, aromatics, fatty acids/esters, nitrogenous compounds, organic acids, sugar alcohols, sugars, terpenes, and steroids. Fatty acids represented the major metabolite class represented by palmitic, stearic, oleic, linoleic, and linolenic acids. Sucrose and pinitol were the major sugars and sugar alcohols among seeds. *Ononis* seeds (OR, OS and OA) were the most abundant in fatty acids, sugars, sugar alcohols, and free amino acids, whereas *Melilotus* species (MO and MS) were least enriched in these key nutrients posing *Ononis* as potential food source for humans and animals. The examined seeds were generally low in sulfur-containing free amino acids and lacking many of the essential free amino acids. Multivariate data analysis aided in the identification of *Ononis* metabolite markers belonging to various classes *i.e.*, (alcohol) glycerol, (sugar) allofuranose, and (sugar alcohol) pinitol, although the differentiation between *Medicago*, *Melilotus*, and *Trifolium* genera was not attained suggestive for other analytical platforms for its classification.

## Introduction

Legume seeds have made a significant contribution to human diet since ancient times being characterized by beneficial nutritional, agricultural, economical, and ecological traits^1^. Legume seeds are considered as a potential source of nutrients including proteins, fibers, vitamins, minerals and carbohydrates^2^ in addition to biologically active compounds (antinutrients) which play an important role in diseases treatment and/or prevention^3^. Members of *Medicago*, *Melilotus*, *Ononis*, and *Trifolium* species F. Fabaceae (Leguminosea) contributed significantly for generations as forage plant in the Mediterranean region^4–6^. *Medicago sativa* (Linn.) seeds the most ancient cultivated fodder plant all over the world, is ranked to be the fourth economically valuable (important) forage crop in North America and temperate regions^7^.

Since ancient times, *Medicago*, *Melilotus*, *Ononis*, and *Trifolium* species were used in traditional medicine. In the Chinese and Hindu societies, physicians make a cooling poultice from *Medicago sativa* seeds commonly known as clover, to be used for boils treatment^7,8^. GC–MS analysis of *M. sativa* seeds revealed its enrichment in crude protein (33.79%), crude oil (8.11%), squalene, hexadecanoic acid methyl ester, n-hexadecanoic acid, 9,12-octadecadienoic acid methyl ester, 9-octadecenamide, and vitamin E^9^. Moreover, *Medicago sativa* seeds inclusion in diet is recommended to normalize serum cholesterol level in type II hyperlipoproteinemia patients^8,10^. Albeit, *Medicago sativa* seed was found to exhibit some health hazards including systemic lupus erythematosus like syndrome in female monkeys^11^ due to its content of canavanine (a nonprotein amino acid), a known anti nutrient.

*Melilotus* species yellow sweet clover was used by Hippocrates and Dioscorides to treat skin ulcers and abscesses due to its emollient and anti-edematous effect^12^, while the Pharaohs used *Melilotus* prepared tea as an anthelmintic^5^. *Melilotus* species are rich in alkaloids, flavonoids, coumarins, triterpenes and saponins^5^. GC–MS Analysis of *n*-hexane extract of *M. officinalis* seed oil revealed the presence of coumarin (hepatotoxic compound) at significant level (8.40%), not recommending its oil utilization for cooking^13^.

Members of the *Ononis* seeds were used internally and externally in ethnomedicine for centuries due to their biologically valuable isoflavonoids and proanthocyanidins content^14,15^. *Ononis natrix* seeds possess a high nutritional value in terms of theoretical nutritional parameters owing to its high protein content (37%), amino acid score (112%), protein efficiency ratio (2.8–2.9), and essential amino acid/ total amino acid (39%)^4^. Previous GC–MS analysis of *O. natrix* seed oil revealed its enrichment in linoleic (33%) and linolenic acids (27%)^16^.

*Trifolium* species were traditionally used as expectorant, analgesic (rheumatic aches), antiseptic and for treatment of constipation, anthelmentic, eczema, psoriasis, lung, nervous and reproductive system disorders^6^. *Trifolium* species are considered as potential source of health phytochemicals due to its high content of quercetin flavonoid and soyasaponin^17^. Furthermore, *Trifolium* seeds were reported to provide the best nutritional values and amino acid composition compared to *Medicago* and *Ononis*^4^.

Due to the increased population worldwide, the demand for the exploration of new healthy alternative for animal proteins and forage plants has increased. Yet the exploration of the safety and nutritional value of *Medicago*, *Melilotus*, *Ononis*, and *Trifolium* species seeds’ as human food has not been fully achieved. Therefore, a comprehensive (integrated) approach for metabolites profiling of 15 leguminous seeds from four different genera (*Medicago*, *Melilotus*, *Ononis*, and *Trifolium*) using gas chromatography–mass spectrometry GC–MS to provide better insight into their primary metabolites content and nutritional traits. Metabolites heterogeneity (diversity) among the different leguminous seeds was measured using unsupervised and supervised multivariate data analysis as principal component analysis (PCA), hierarchical cluster analysis (HCA) and orthogonal partial least squares discriminant analysis (OPLS-DA) and to aid identify markers of each genus.

## Materials and methods

### Plant material

The dried legume seeds viz. *Melilotus*, *Trifolium*, *Medicago*, and *Ononis* different species were obtained with permission from the Department for Bioarchaeology, Austrian Archaeological Institute (OeAI), Austrian Academy of Sciences (OeAW), Austria (Table 1). The experimental study of the seeds complied with all the appropriate guideline^18^. Voucher specimens were kept at the Herbarium of Faculty of Pharmacy, Cairo University, Cairo, Egypt. Analysis of each sample was carried out in triplicate to consider the biological variation.

**Table 1:** Sample codes of legume seed species used in this study.

Sample code	Latin plant name
MA	*Melilotus albus*
MO	*Melilotus officinalis*
MS	*Melilotus segetalis*
TP	*Trifolium pannonic*
TI	*Trifolium incarnatu*
TM	*Trifolium montanu*
TA	*Trifolium arvense*
MDS	*Medicago sativa*
MDO	*Medicago orbiculari*
ML	*Medicago lupulina*
MX	*Medicago xvaria*
OR	*Ononis repens*
ON	*Ononis natrix*
OS	*Ononis spinosa*
OA	*Ononis arvensis*

### GC–MS analysis of the silylated primary metabolites

The analysis of primary metabolites was conducted as described in^19^. In brief, the finely powdered seeds (100 mg) were extracted with methanol and centrifugated at 12,000 rpm for 10 min to get rid of the debris. Samples were sonicated and extracted once, following the same protocol as^20,21^.

Three samples of each seed were analysed using the same conditions to consider the biological variation. The methanol extracts were evaporated under a nitrogen gas stream till dryness. Dried pellet was derivatized using 150 µL of *N*-methyl-*N*-(trimethylsilyl)-trifluoroacetamide (MSTFA) and incubated for 45 min at 60 °C. GC/MS analysis was carried out on a Shimadzu GC-17A gas chromatograph that is coupled to Shimadzu QP5050A mass spectrometer, using Rtx-5MS column (30 m length, 0.25 mm inner diameter, and 0.25 μm film thickness).

### Validation and quality control of samples for nutrient analysis using GC–MS

Three pooled quality control samples were injected before GC–MS analysis. The pooled quality control samples were injected multiple times during the whole experiment to further ensure the stability and accuracy of the analysis^20,21^. The relative standard deviation (RSD) of retention time was in the range of 0.05–0.15%. The RSD of peak intensity varied between 2.63 and 8.08%.

For metabolites quantification, soluble sugars, free amino acids, organic acids and fatty acids were quantified using standard curves of glucose, glycine, citric and stearic acids and results were expressed as mg/g. Four serial dilutions were prepared from 10 to 600 μg/mL for establishing the standard curves. Calibration curves for glucose, glycine, citric acid and stearic acids displayed 0.9948 correlation coefficient.

### Metabolites identification and absolute quantification

First GC/MS peaks were deconvoluted using AMDIS software (https://www.amdis.net), afterwards the identification of silylated metabolites was accomplished by comparison of their retention indices (RI) relative to n-alkanes series (C8–C30), and their mass spectra matching to WILEY, NIST library databases and also with standards whenever available. Peak abundance was obtained using MS-DIAL software with previously described parameters in^22^ Alcohols, organic acids, fatty acids, soluble sugars and free amino acids were quantified using the standard curves of glycerol, lactic acid, stearic acid, glucose and glycine and expressed as mg/g. For the standard curves, four serial dilutions were prepared (from 10 to 600 µg/mL). Calibration curves for glucose, glycine, and stearic acids displayed a correlation coefficient of ca. 0.9948^21^.

### Multivariate data analysis

The multivariate data analysis (MVDA) was carried out using both the unsupervised principal component analysis (PCA) and hierarchical cluster analysis (HCA), in addition to the supervised orthogonal partial least squares-discriminate analysis (OPLS-DA) using SIMCA 14.1 (Umetrics, Umea, Sweden), all variables were scaled and mean centered to Pareto Variance. The unsupervised PCA was performed for acquiring an extensive configuration (overview or figure) of the variance of metabolites among the different seeds’ specimens, while the supervised OPLS-DA was implemented to confirm PCA results and to access detailed information on the distinctions (variation or differences) in metabolites composition among the studied specimens. Chemometric models were assessed utilizing the two parameters (specifications) R^2^ and Q^2^ number of permutations in models set at 200. R^2^ was employed to specify the model goodness of fit, while Q^2^ indicated the model predictability. Outliers were detected using DModx (distance to the model) whereas strong outliers’ detection for the OPLS-DA plot was performed using Hotelling’s T2. An iterative permutation test was carried out to eradicate the non-randomness of separation among groups.

### Enrichment analysis

Enrichment analysis was performed using MetaboAnalyst 5.0 (https://www.metaboanalyst.ca, accessed on 17 September 2023) by annotating KEGG IDs with main-class and “sub-class” metabolite chemical sets.

## Results and discussion

The main goal of this study was to evaluate metabolome diversity within less explored legume seed *species.* The examined samples comprised three *Melilotus*, four *Medicago*, four *Trifolium*, and four *Ononis* seed species represented by different species (Table 1). To assess the biological variance within each sample as well as the analysis conditions, three independent biological specimens were analyzed using GC–MS.

### GC/MS-based metabolite profiling

**Figure 1:**
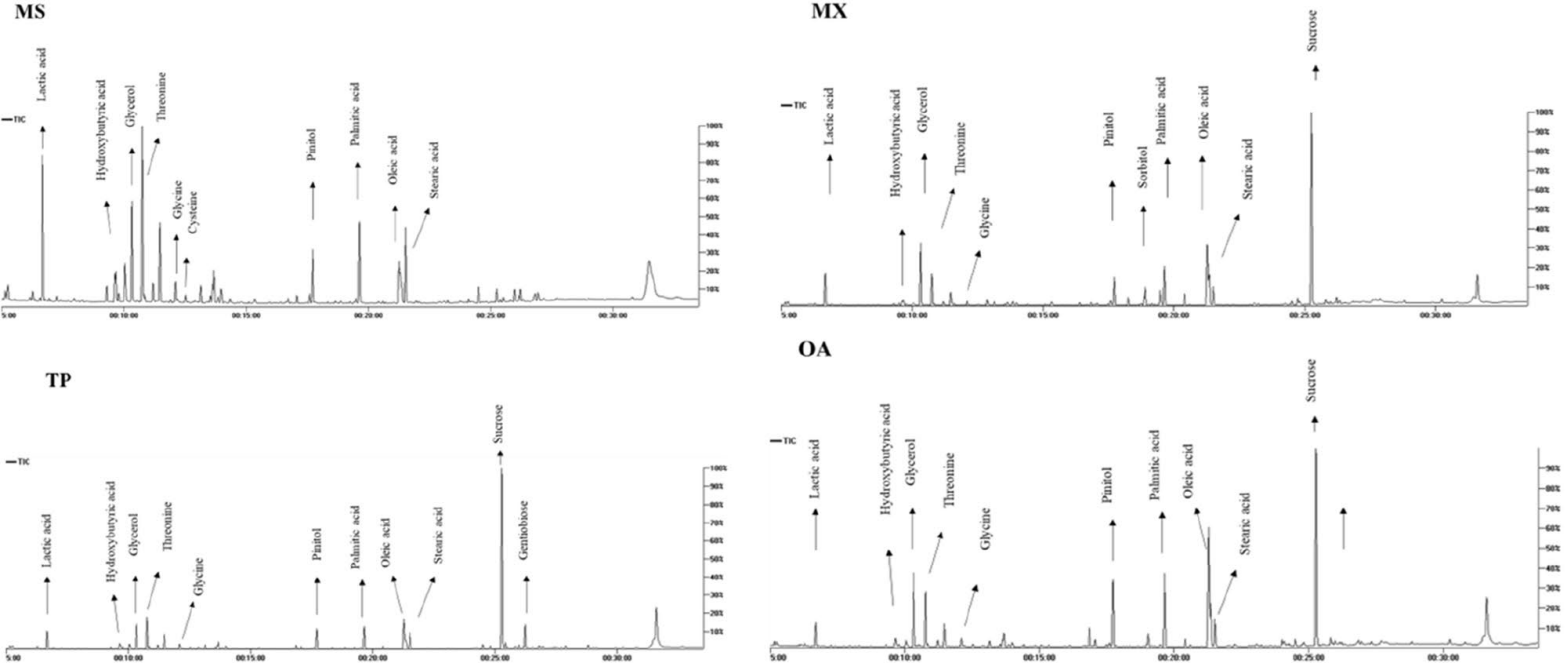
Representative GC–MS chromatograms of TMS derivatives of metabolites in the extracts of “MS” *Melilotus segetalis*, “MX” *Medicago xvaria*, “TP” *Trifolium pannonic*, “OA” *Ononis arvensis.*

**Figure 2:**
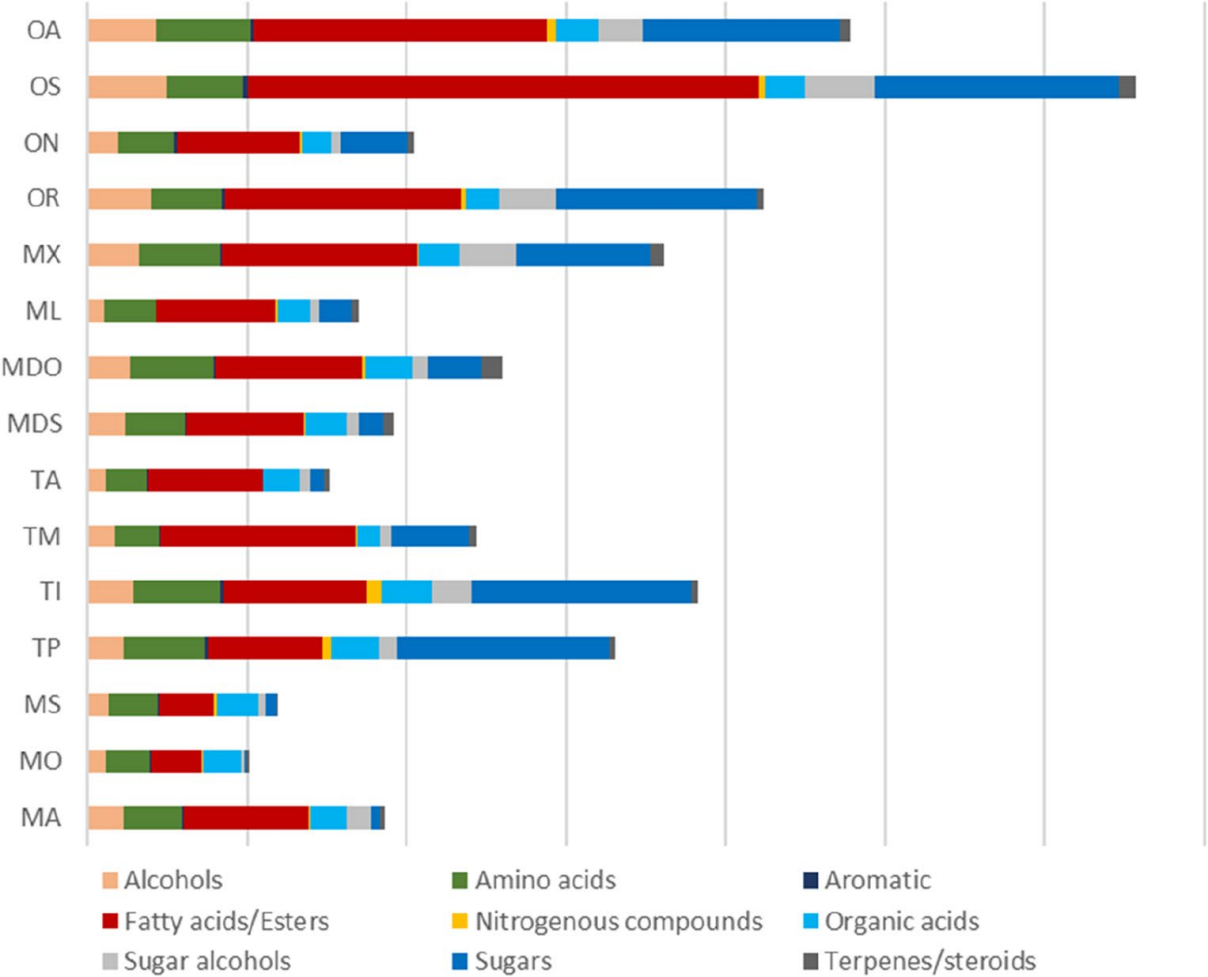
A bar chart illustrating the concentration of major metabolite classes in *Melilotus, Trifolium, Medicago,* and *Ononis* seed species expressed as mg/g. MA, *Melilotus albus*; MO, *Melilotus officinalis*; MS, *Melilotus segetalis*; TP, *Trifolium pannonic*; TI, *Trifolium incarnatu*; TM, *Trifolium montanu*; TA, *Trifolium arvense*; MDS, *Medicago sativa*; MDO, *Medicago orbiculari*; ML, *Medicago lupulina*; MX, *Medicago xvaria*; OR, *Ononis repens*; ON, *Ononis natrix*; OS, *Ononis spinosa*; OA, *Ononis arvensis*.

GC–MS analysis was carried out post-silylation to assess seeds’ metabolome in context to its low molecular weight primary metabolites, Fig. 1**.** About 87 compounds **(**Table 2**)** were identified, comprising alcohols, amino acids, aromatics, fatty acids/esters, nitrogenous compounds, organic acids, sugar alcohols, sugars, terpenes, and steroids. The major annotated metabolites among all examined seeds are represented in Fig. 2**.**

**Table 2:** Levels of silylated primary metabolites in *Melilotus, Trifolium, Medicago*, and *Ononis* seed species.

Peak no	Average Rt (min)	KI	Name	MA	MO	MS	TP	TI	TM	TA	MDS	MDO	ML	MX	OR	ON	OS	OA
Alcohols																		
1	5.27	988.2	Ethylene glycol, (2TMS)	0.55 ± 0.03	0.51 ± 0.03	0.51 ± 0.02	0.58 ± 0.03	0.64 ± 0.05	0.51 ± 0.01	0.52 ± 0.01	0.56 ± 0.02	0.60 ± 0.02	0.50 ± 0.01	0.55 ± 0.04	0.48 ± 0.02	0.50 ± 0.02	0.60 ± 0.19	0.74 ± 0.05
2	6.56	1058.6	1,3 Propanediol, (2TMS)	0.11 ± 0.01	0.10 ± 0.01	0.10 ± 0.00	0.11 ± 0.01	0.14 ± 0.01	0.10 ± 0.01	0.10 ± 0.00	0.07 ± 0.02	0.12 ± 0.02	0.09 ± 0.01	0.11 ± 0.00	0.10 ± 0.01	0.11 ± 0.01	0.13 ± 0.03	0.16 ± 0.01
3	10.33	1281.7	Glycerol, (3TMS)	3.78 ± 1.69	1.48 ± 0.82	1.97 ± 0.38	3.69 ± 0.42	4.80 ± 1.64	2.61 ± 2.77	1.47 ± 0.64	3.98 ± 1.79	4.56 ± 1.39	1.39 ± 0.19	5.71 ± 3.29	7.36 ± 2.32	3.02 ± 0.33	9.04 ± 3.05	7.44 ± 1.98
4	11.50	1357.8	1-Decanol, O-TMS	0.02 ± 0.01	0.03 ± 0.01	0.03 ± 0.00	0.03 ± 0.01	0.04 ± 0.00	0.02 ± 0.00	0.03 ± 0.01	0.03 ± 0.01	0.03 ± 0.02	0.02 ± 0.00	0.02 ± 0.00	0.02 ± 0.00	0.02 ± 0.00	0.03 ± 0.01	0.04 ± 0.00
5	14.51	1578.6	Alpha-hydroxyglutaric acid (3TMS)	0.01 ± 0.00	0.00 ± 0.00	0.00 ± 0.00	0.01 ± 0.00	0.01 ± 0.00	0.00 ± 0.00	0.00 ± 0.00	0.01 ± 0.00	0.02 ± 0.00	0.00 ± 0.00	0.01 ± 0.00	0.01 ± 0.00	0.01 ± 0.00	0.01 ± 0.00	0.01 ± 0.00
6	20.70	2144.5	1-Octadecanol, O-TMS	0.03 ± 0.00	0.03 ± 0.00	0.03 ± 0.00	0.05 ± 0.02	0.04 ± 0.00	0.03 ± 0.01	0.03 ± 0.00	0.07 ± 0.04	0.03 ± 0.00	0.04 ± 0.00	0.04 ± 0.00	0.04 ± 0.01	0.07 ± 0.05	0.06 ± 0.02	0.05 ± 0.00
7	21.20	2196.5	1-Hexadecanol, DMTBS	0.01 ± 0.00	0.00 ± 0.00	0.01 ± 0.00	0.01 ± 0.00	0.02 ± 0.03	0.01 ± 0.01	0.01 ± 0.01	0.01 ± 0.00	0.01 ± 0.00	0.01 ± 0.00	0.01 ± 0.00	0.01 ± 0.00	0.01 ± 0.00	0.02 ± 0.02	0.02 ± 0.00
Total alcohols				4.51	2.15	2.63	4.48	5.70	3.28	2.17	4.72	5.37	2.05	6.45	8.02	3.74	9.90	8.46
Amino acids																		
8	7.11	1088.8	Valine, TMS	0.16 ± 0.23	0.01 ± 0.00	0.01 ± 0.01	0.07 ± 0.03	0.03 ± 0.02	0.02 ± 0.03	0.02 ± 0.01	0.01 ± 0.01	0.03 ± 0.02	0.02 ± 0.00	0.08 ± 0.08	0.06 ± 0.00	0.03 ± 0.02	0.06 ± 0.02	0.09 ± 0.01
9	7.43	1106.1	Alanine, (2TMS)	0.23 ± 0.37	0.01 ± 0.00	0.01 ± 0.00	0.14 ± 0.08	0.10 ± 0.10	0.03 ± 0.05	0.01 ± 0.00	0.02 ± 0.01	0.06 ± 0.04	0.01 ± 0.00	0.12 ± 0.09	0.19 ± 0.12	0.03 ± 0.04	0.12 ± 0.04	0.18 ± 0.03
10	8.51	1166.6	1-Methylproline	0.04 ± 0.01	0.02 ± 0.00	0.01 ± 0.00	0.04 ± 0.01	0.08 ± 0.10	0.03 ± 0.01	0.02 ± 0.00	0.10 ± 0.07	0.96 ± 0.57	0.06 ± 0.02	0.76 ± 0.41	0.03 ± 0.01	0.03 ± 0.01	0.04 ± 0.00	0.03 ± 0.01
11	8.66	1175.4	Isoleucine, TMS	0.07 ± 0.10	0.01 ± 0.00	0.01 ± 0.00	0.03 ± 0.01	0.01 ± 0.00	0.01 ± 0.01	0.01 ± 0.00	0.01 ± 0.01	0.01 ± 0.01	0.01 ± 0.01	0.03 ± 0.02	0.02 ± 0.00	0.01 ± 0.00	0.03 ± 0.02	0.04 ± 0.01
12	9.37	1217.4	Valine, (2TMS)	0.17 ± 0.22	0.00 ± 0.00	0.01 ± 0.01	0.13 ± 0.07	0.06 ± 0.05	0.03 ± 0.04	0.01 ± 0.01	0.03 ± 0.03	0.03 ± 0.03	0.02 ± 0.00	0.06 ± 0.06	0.09 ± 0.04	0.03 ± 0.02	0.08 ± 0.00	0.14 ± 0.02
13	10.01	1260.5	Serine, (2TMS)	0.02 ± 0.02	0.02 ± 0.02	0.03 ± 0.01	0.02 ± 0.01	0.03 ± 0.02	0.05 ± 0.02	0.02 ± 0.02	0.42 ± 0.37	0.48 ± 0.36	0.76 ± 0.09	0.30 ± 0.10	0.04 ± 0.01	0.04 ± 0.01	0.06 ± 0.03	0.06 ± 0.03
14	10.26	1276.5	Leucine, (2TMS)	0.19 ± 0.27	0.00 ± 0.00	0.01 ± 0.00	0.06 ± 0.02	0.04 ± 0.01	0.02 ± 0.04	0.01 ± 0.01	0.02 ± 0.03	0.03 ± 0.01	0.02 ± 0.00	0.06 ± 0.07	0.09 ± 0.04	0.03 ± 0.01	0.07 ± 0.01	0.12 ± 0.01
15	10.77	1299.3	l-Threonine, (2TMS)	3.66 ± 0.27	3.65 ± 0.22	3.57 ± 0.07	4.04 ± 0.19	4.95 ± 0.32	3.42 ± 0.08	3.42 ± 0.15	4.32 ± 0.23	4.49 ± 0.13	3.36 ± 0.04	3.73 ± 0.26	3.49 ± 0.20	3.62 ± 0.19	4.36 ± 0.63	5.24 ± 0.05
16	12.03	1392	Homoserine, (3TMS)	0.04 ± 0.03	0.01 ± 0.00	0.01 ± 0.01	0.04 ± 0.01	0.04 ± 0.02	0.01 ± 0.01	0.01 ± 0.00	0.01 ± 0.01	0.02 ± 0.01	0.01 ± 0.00	0.03 ± 0.02	0.03 ± 0.01	0.01 ± 0.00	0.02 ± 0.00	0.04 ± 0.01
17	12.11	1398	Glycine, (3TMS)	1.73 ± 0.23	1.62 ± 0.13	1.73 ± 0.09	2.23 ± 0.19	2.87 ± 0.20	1.41 ± 0.33	1.23 ± 0.20	1.64 ± 1.24	2.41 ± 0.06	1.45 ± 0.11	1.81 ± 0.11	1.89 ± 0.05	1.93 ± 0.14	2.59 ± 0.65	3.25 ± 0.07
18	12.47	1425.2	Aspartic acid, (2TMS)	0.01 ± 0.01	0.01 ± 0.01	0.01 ± 0.00	0.07 ± 0.04	0.06 ± 0.04	0.01 ± 0.02	0.01 ± 0.01	0.01 ± 0.00	0.03 ± 0.03	0.01 ± 0.00	0.07 ± 0.03	0.17 ± 0.21	0.03 ± 0.03	0.11 ± 0.10	0.04 ± 0.02
19	12.88	1455.9	Cysteine, 3(TMS)	0.06 ± 0.05	0.01 ± 0.00	0.01 ± 0.00	1.87 ± 1.35	0.05 ± 0.04	0.14 ± 0.24	0.02 ± 0.01	0.09 ± 0.07	0.07 ± 0.03	0.16 ± 0.06	1.11 ± 0.85	0.15 ± 0.08	0.11 ± 0.09	0.18 ± 0.04	0.33 ± 0.10
20	13.83	1527.6	l-Aspartic acid, (3TMS)-	0.03 ± 0.03	0.01 ± 0.00	0.03 ± 0.01	0.18 ± 0.09	0.28 ± 0.18	0.06 ± 0.09	0.02 ± 0.01	0.03 ± 0.02	0.13 ± 0.13	0.05 ± 0.01	0.17 ± 0.05	0.72 ± 0.89	0.16 ± 0.15	0.38 ± 0.21	0.17 ± 0.09
21	13.86	1529.7	Pyroglutamic acid, (N,O-TMS)	0.78 ± 0.46	0.26 ± 0.12	0.56 ± 0.07	0.99 ± 0.23	1.92 ± 0.97	0.32 ± 0.28	0.38 ± 0.25	0.58 ± 0.46	1.15 ± 0.12	0.35 ± 0.09	1.34 ± 0.85	1.26 ± 0.17	0.71 ± 0.39	1.17 ± 0.31	1.49 ± 0.20
22	15.07	1623.6	Glutamic acid (3TMS)	0.03 ± 0.02	0.01 ± 0.00	0.01 ± 0.00	0.19 ± 0.11	0.25 ± 0.25	0.05 ± 0.07	0.01 ± 0.01	0.02 ± 0.01	0.22 ± 0.10	0.04 ± 0.00	0.16 ± 0.09	0.36 ± 0.05	0.19 ± 0.14	0.23 ± 0.06	0.52 ± 0.22
23	15.19	1633.2	Phenylalanine (2TMS)	0.04 ± 0.06	0.01 ± 0.00	0.01 ± 0.00	0.06 ± 0.01	0.03 ± 0.01	0.03 ± 0.04	0.01 ± 0.00	0.01 ± 0.01	0.04 ± 0.02	0.02 ± 0.01	0.06 ± 0.06	0.06 ± 0.04	0.04 ± 0.03	0.06 ± 0.02	0.10 ± 0.03
24	18.70	1947.4	Tyrosine, (3TMS)	0.07 ± 0.08	0.01 ± 0.00	0.02 ± 0.00	0.09 ± 0.01	0.11 ± 0.05	0.03 ± 0.03	0.02 ± 0.01	0.06 ± 0.04	0.22 ± 0.10	0.05 ± 0.02	0.22 ± 0.25	0.07 ± 0.03	0.05 ± 0.02	0.09 ± 0.01	0.11 ± 0.01
Total amino acids				7.33	5.66	6.05	10.22	10.89	5.68	5.22	7.38	10.39	6.39	10.11	8.71	7.03	9.64	11.96
Aromatic																		
25	9.81	1246.6	Benzoic acid, TMS	0.07 ± 0.05	0.10 ± 0.06	0.06 ± 0.01	0.14 ± 0.05	0.32 ± 0.23	0.04 ± 0.00	0.11 ± 0.07	0.06 ± 0.02	0.11 ± 0.09	0.05 ± 0.00	0.08 ± 0.04	0.24 ± 0.03	0.13 ± 0.04	0.30 ± 0.02	0.21 ± 0.05
26	13.79	1524.8	4-methoxybenzoic acid (TMS)	0.06 ± 0.01	0.06 ± 0.00	0.06 ± 0.00	0.08 ± 0.00	0.08 ± 0.02	0.05 ± 0.01	0.06 ± 0.01	0.08 ± 0.00	0.07 ± 0.01	0.05 ± 0.00	0.05 ± 0.01	0.06 ± 0.00	0.06 ± 0.01	0.06 ± 0.01	0.08 ± 0.01
27	15.13	1628.3	*p*-Hydroxybenzoic acid (TMS)	0.04 ± 0.01	0.02 ± 0.00	0.02 ± 0.01	0.05 ± 0.00	0.05 ± 0.02	0.03 ± 0.01	0.03 ± 0.00	0.03 ± 0.01	0.07 ± 0.01	0.04 ± 0.00	0.04 ± 0.01	0.03 ± 0.00	0.12 ± 0.04	0.03 ± 0.00	0.03 ± 0.00
28	18.67	1941.4	*p*-Coumaric acid (2TMS)	0.03 ± 0.01	0.01 ± 0.00	0.01 ± 0.00	0.02 ± 0.00	0.02 ± 0.00	0.02 ± 0.01	0.01 ± 0.00	0.02 ± 0.01	0.02 ± 0.00	0.02 ± 0.00	0.03 ± 0.01	0.02 ± 0.00	0.05 ± 0.01	0.02 ± 0.00	0.02 ± 0.00
Total aromatic				0.21	0.19	0.15	0.28	0.47	0.14	0.21	0.19	0.27	0.15	0.21	0.34	0.36	0.41	0.34
Fatty acids/esters																		
29	15.72	1647.1	Lauric acid, TMS	0.03 ± 0.04	0.01 ± 0.01	0.04 ± 0.01	0.26 ± 0.17	0.20 ± 0.08	0.09 ± 0.12	0.03 ± 0.01	0.07 ± 0.04	0.30 ± 0.18	0.07 ± 0.01	0.13 ± 0.04	0.91 ± 1.31	0.13 ± 0.08	0.25 ± 0.19	0.13 ± 0.01
30	17.60	1840.9	Myristic acid, TMS	0.68 ± 0.04	0.35 ± 0.04	0.36 ± 0.02	0.47 ± 0.03	0.59 ± 0.08	1.29 ± 0.05	1.16 ± 0.03	1.27 ± 0.25	0.81 ± 0.08	1.09 ± 0.03	1.07 ± 0.08	0.54 ± 0.02	0.64 ± 0.06	0.56 ± 0.17	0.63 ± 0.03
31	18.44	1919.1	Palmitic acid, methyl ester	0.05 ± 0.01	0.03 ± 0.02	0.02 ± 0.00	0.06 ± 0.00	0.06 ± 0.01	0.04 ± 0.02	0.04 ± 0.02	0.06 ± 0.01	0.06 ± 0.00	0.05 ± 0.00	0.06 ± 0.01	0.06 ± 0.01	0.09 ± 0.03	0.08 ± 0.02	0.09 ± 0.02
32	18.65	1939.3	Pentadecanoic acid, TMS ester	0.10 ± 0.02	0.08 ± 0.02	0.08 ± 0.00	0.13 ± 0.02	0.17 ± 0.07	0.08 ± 0.02	0.09 ± 0.02	0.27 ± 0.09	0.14 ± 0.03	0.10 ± 0.01	0.14 ± 0.05	0.13 ± 0.01	0.13 ± 0.04	0.16 ± 0.04	0.21 ± 0.02
33	19.65	2037.9	Palmitic acid, TMS	4.19 ± 1.16	1.90 ± 0.18	2.30 ± 0.27	4.04 ± 0.23	6.03 ± 1.17	5.63 ± 1.53	4.37 ± 0.67	5.32 ± 0.75	6.14 ± 0.84	4.50 ± 0.26	6.83 ± 1.69	6.81 ± 0.82	4.35 ± 0.86	10.51 ± 3.73	9.94 ± 0.76
34	20.60	2134	Margaric acid, TMS	0.12 ± 0.02	0.08 ± 0.00	0.09 ± 0.01	0.11 ± 0.01	0.15 ± 0.04	0.14 ± 0.00	0.12 ± 0.01	0.17 ± 0.00	0.14 ± 0.01	0.12 ± 0.01	0.14 ± 0.02	0.14 ± 0.02	0.13 ± 0.02	0.30 ± 0.22	0.19 ± 0.01
35	21.28	2204	Oleic acid, TMS	2.95 ± 2.18	0.63 ± 0.01	0.83 ± 0.15	3.82 ± 0.61	3.07 ± 0.60	10.02 ± 7.67	4.13 ± 0.96	2.44 ± 0.70	4.08 ± 2.20	4.38 ± 0.39	7.56 ± 2.63	9.86 ± 3.25	3.54 ± 1.90	27.29 ± 28.40	12.53 ± 1.57
36	21.246	2205.3	Linoleic acid, TMS	0.85 ± 0.91	0.04 ± 0.01	0.20 ± 0.12	1.07 ± 0.18	1.04 ± 0.24	1.83 ± 2.34	0.34 ± 0.17	0.19 ± 0.10	1.00 ± 0.84	0.39 ± 0.08	1.98 ± 0.90	3.47 ± 1.10	1.12 ± 0.51	3.35 ± 2.63	4.08 ± 0.30
37	21.342	2213	α-Linolenic acid, TMS	2.03 ± 1.98	0.12 ± 0.06	0.28 ± 0.11	0.52 ± 0.22	0.52 ± 0.17	0.88 ± 0.31	0.39 ± 0.12	0.55 ± 0.17	1.19 ± 0.78	0.51 ± 0.14	2.16 ± 0.62	3.09 ± 1.13	1.69 ± 0.91	3.42 ± 1.51	2.98 ± 0.44
38	21.52	2231.3	Stearic acid, TMS	2.66 ± 0.19	2.12 ± 0.14	2.09 ± 0.05	2.76 ± 0.05	4.18 ± 0.62	3.34 ± 0.69	2.61 ± 0.16	3.01 ± 0.41	2.87 ± 0.18	2.66 ± 0.08	3.01 ± 0.35	3.06 ± 0.48	2.51 ± 0.18	9.24 ± 9.01	3.81 ± 0.35
39	23.24	2426.2	Arachidic acid, TMS	0.19 ± 0.05	0.08 ± 0.01	0.09 ± 0.01	0.14 ± 0.02	0.39 ± 0.16	0.30 ± 0.05	0.20 ± 0.01	0.24 ± 0.09	0.28 ± 0.05	0.24 ± 0.03	0.29 ± 0.06	0.39 ± 0.12	0.18 ± 0.03	2.13 ± 2.73	0.31 ± 0.09
40	24.05	2529.4	Heneicosanoic acid, TMS	0.02 ± 0.01	0.01 ± 0.01	0.02 ± 0.01	0.03 ± 0.00	0.11 ± 0.10	0.06 ± 0.01	0.04 ± 0.01	0.04 ± 0.01	0.04 ± 0.00	0.03 ± 0.00	0.06 ± 0.01	0.10 ± 0.00	0.05 ± 0.01	0.33 ± 0.39	0.12 ± 0.10
41	24.50	2587.1	1-Monopalmitin, TMS	1.59 ± 0.50	0.68 ± 0.47	0.45 ± 0.07	0.76 ± 0.12	0.92 ± 0.24	0.48 ± 0.15	0.48 ± 0.14	0.79 ± 0.14	1.00 ± 0.17	0.50 ± 0.03	0.66 ± 0.16	0.82 ± 0.08	0.59 ± 0.13	3.97 ± 5.04	1.37 ± 0.37
42	24.84	2629.9	Behenic acid, TMS	0.12 ± 0.01	0.05 ± 0.00	0.06 ± 0.01	0.12 ± 0.03	0.39 ± 0.18	0.19 ± 0.04	0.14 ± 0.02	0.17 ± 0.08	0.22 ± 0.04	0.17 ± 0.02	0.26 ± 0.08	0.37 ± 0.07	0.17 ± 0.03	1.70 ± 2.27	0.30 ± 0.08
43	26.32	2813.9	Lignoceric acid, TMS	0.09 ± 0.01	0.04 ± 0.01	0.04 ± 0.00	0.07 ± 0.01	0.11 ± 0.05	0.13 ± 0.01	0.12 ± 0.01	0.12 ± 0.04	0.12 ± 0.02	0.11 ± 0.01	0.12 ± 0.02	0.08 ± 0.01	0.09 ± 0.01	0.92 ± 1.39	0.15 ± 0.02
Total fatty acids/esters				15.67	6.24	6.96	14.36	17.92	24.51	14.25	14.72	18.40	14.93	24.46	29.84	15.40	64.20	36.83
Nitrogenous compounds																		
44	10.54	1294.8	Nicotinic acid, TMS	0.05 ± 0.01	0.02 ± 0.00	0.22 ± 0.16	0.94 ± 0.36	1.51 ± 0.67	0.06 ± 0.06	0.05 ± 0.01	0.08 ± 0.07	0.09 ± 0.03	0.17 ± 0.04	0.15 ± 0.06	0.26 ± 0.06	0.17 ± 0.08	0.48 ± 0.10	0.66 ± 0.17
45	15.14	1629	Triethanolamine, (3TMS)	0.11 ± 0.10	0.15 ± 0.15	0.11 ± 0.04	0.10 ± 0.04	0.14 ± 0.08	0.02 ± 0.01	0.05 ± 0.04	0.15 ± 0.12	0.08 ± 0.06	0.04 ± 0.02	0.05 ± 0.04	0.04 ± 0.02	0.05 ± 0.01	0.04 ± 0.02	0.05 ± 0.02
46	23.19	2420.2	Uridine, (3 O-TMS)	0.01 ± 0.01	0.01 ± 0.00	0.01 ± 0.02	0.02 ± 0.01	0.04 ± 0.03	0.04 ± 0.04	0.02 ± 0.02	0.03 ± 0.03	0.03 ± 0.01	0.01 ± 0.00	0.03 ± 0.01	0.02 ± 0.00	0.01 ± 0.01	0.03 ± 0.02	0.06 ± 0.00
47	23.58	2468.8	Uridine, (3 O-TMS)	0.04 ± 0.03	0.01 ± 0.00	0.01 ± 0.01	0.13 ± 0.03	0.20 ± 0.16	0.06 ± 0.06	0.02 ± 0.01	0.01 ± 0.00	0.07 ± 0.01	0.04 ± 0.00	0.07 ± 0.01	0.12 ± 0.02	0.09 ± 0.05	0.21 ± 0.09	0.27 ± 0.04
Total nitrogenous compounds				0.21	0.19	0.36	1.19	1.90	0.18	0.14	0.28	0.27	0.26	0.30	0.44	0.32	0.77	1.04
Organic acids																		
48	5.56	1004.1	Lactic acid, (2TMS)	0.04 ± 0.00	0.04 ± 0.00	0.04 ± 0.00	0.05 ± 0.01	0.05 ± 0.00	0.04 ± 0.00	0.04 ± 0.00	0.05 ± 0.00	0.04 ± 0.00	0.04 ± 0.00	0.04 ± 0.00	0.04 ± 0.00	0.04 ± 0.00	0.05 ± 0.01	0.06 ± 0.01
49	6.38	1048.6	Glycolic acid, (2TMS)	0.05 ± 0.01	0.05 ± 0.00	0.05 ± 0.00	0.06 ± 0.00	0.07 ± 0.01	0.05 ± 0.00	0.05 ± 0.00	0.06 ± 0.01	0.06 ± 0.00	0.05 ± 0.01	0.06 ± 0.00	0.05 ± 0.00	0.05 ± 0.01	0.07 ± 0.01	0.08 ± 0.00
50	6.69	1065.9	Lactic acid, (2TMS)	2.11 ± 1.00	2.45 ± 1.24	3.00 ± 0.71	2.82 ± 0.31	2.93 ± 0.62	1.16 ± 0.60	2.61 ± 1.88	2.45 ± 1.40	2.97 ± 0.47	1.91 ± 0.41	2.58 ± 1.70	1.88 ± 0.71	1.37 ± 0.43	1.43 ± 0.29	1.97 ± 0.87
51	6.83	1073.1	Caproic acid (TMS)	0.02 ± 0.00	0.02 ± 0.00	0.02 ± 0.00	0.04 ± 0.00	0.02 ± 0.01	0.03 ± 0.01	0.02 ± 0.00	0.04 ± 0.02	0.03 ± 0.01	0.03 ± 0.01	0.03 ± 0.01	0.04 ± 0.01	0.03 ± 0.01	0.06 ± 0.03	0.05 ± 0.01
52	6.95	1079.7	Glycolic acid, (2TMS)	0.14 ± 0.02	0.11 ± 0.03	0.11 ± 0.02	0.16 ± 0.02	0.22 ± 0.03	0.09 ± 0.01	0.12 ± 0.01	0.19 ± 0.06	0.20 ± 0.01	0.14 ± 0.02	0.13 ± 0.04	0.14 ± 0.03	0.12 ± 0.02	0.16 ± 0.04	0.18 ± 0.02
53	7.16	1091.4	Pyruvic acid, (2TMS)	0.04 ± 0.02	0.02 ± 0.00	0.02 ± 0.00	0.03 ± 0.00	0.04 ± 0.01	0.02 ± 0.00	0.02 ± 0.00	0.08 ± 0.02	0.04 ± 0.02	0.03 ± 0.01	0.03 ± 0.00	0.03 ± 0.01	0.03 ± 0.00	0.04 ± 0.01	0.04 ± 0.00
54	8.14	1146.1	β-Lactic acid, (2TMS)	0.11 ± 0.06	0.08 ± 0.02	0.06 ± 0.01	0.12 ± 0.02	0.13 ± 0.02	0.05 ± 0.01	0.08 ± 0.01	0.09 ± 0.01	0.11 ± 0.04	0.07 ± 0.01	0.08 ± 0.02	0.08 ± 0.03	0.06 ± 0.01	0.09 ± 0.01	0.10 ± 0.01
55	8.37	1162	Malonic acid, (2TMS)	0.04 ± 0.03	0.02 ± 0.00	0.02 ± 0.00	0.03 ± 0.00	0.01 ± 0.01	0.01 ± 0.01	0.02 ± 0.00	0.13 ± 0.14	0.03 ± 0.01	0.02 ± 0.01	0.01 ± 0.01	0.01 ± 0.01	0.01 ± 0.01	0.02 ± 0.01	0.02 ± 0.01
56	8.52	1167.4	2-Hydroxyvaleric acid, (2TMS)	0.01 ± 0.00	0.01 ± 0.00	0.01 ± 0.00	0.01 ± 0.00	0.01 ± 0.00	0.01 ± 0.00	0.01 ± 0.00	0.01 ± 0.00	0.01 ± 0.00	0.01 ± 0.00	0.01 ± 0.00	0.04 ± 0.00	0.01 ± 0.00	0.05 ± 0.02	0.03 ± 0.01
57	9.17	1204.4	Malonic acid, (2TMS)	0.01 ± 0.01	0.01 ± 0.01	0.01 ± 0.01	0.01 ± 0.00	0.01 ± 0.01	0.01 ± 0.00	0.01 ± 0.00	0.01 ± 0.00	0.01 ± 0.01	0.01 ± 0.00	0.03 ± 0.01	0.03 ± 0.01	0.03 ± 0.02	0.04 ± 0.03	0.03 ± 0.02
58	9.64	1235.6	γ-Hydroxybutyric acid, (2TMS)	1.02 ± 0.09	1.10 ± 0.20	0.95 ± 0.03	1.26 ± 0.26	1.40 ± 0.30	0.70 ± 0.04	0.76 ± 0.06	0.99 ± 0.11	1.09 ± 0.04	0.75 ± 0.01	0.94 ± 0.12	0.84 ± 0.04	1.02 ± 0.13	1.28 ± 0.48	1.36 ± 0.09
59	10.04	1261.9	Octanoic acid, TMS ester	0.02 ± 0.02	0.04 ± 0.01	0.03 ± 0.00	0.03 ± 0.00	0.04 ± 0.01	0.03 ± 0.00	0.03 ± 0.01	0.04 ± 0.01	0.04 ± 0.01	0.03 ± 0.00	0.02 ± 0.01	0.03 ± 0.00	0.03 ± 0.00	0.05 ± 0.01	0.05 ± 0.00
60	10.86	1315.6	Succinic acid, (2TMS)	0.34 ± 0.03	0.28 ± 0.03	0.28 ± 0.00	0.42 ± 0.05	0.48 ± 0.03	0.27 ± 0.02	0.28 ± 0.02	0.38 ± 0.02	0.44 ± 0.01	0.30 ± 0.01	0.34 ± 0.05	0.32 ± 0.02	0.31 ± 0.05	0.43 ± 0.05	0.50 ± 0.01
61	11.21	1338.3	Glyceric acid, (3TMS)	0.42 ± 0.10	0.37 ± 0.02	0.37 ± 0.00	0.45 ± 0.01	0.54 ± 0.03	0.32 ± 0.01	0.32 ± 0.02	0.45 ± 0.08	0.49 ± 0.04	0.34 ± 0.02	0.38 ± 0.03	0.37 ± 0.03	0.38 ± 0.04	0.47 ± 0.06	0.59 ± 0.01
62	11.35	1346.9	Fumaric acid, (2TMS)	0.01 ± 0.01	0.00 ± 0.00	0.01 ± 0.01	0.02 ± 0.00	0.02 ± 0.00	0.01 ± 0.01	0.00 ± 0.00	0.03 ± 0.02	0.05 ± 0.01	0.03 ± 0.01	0.03 ± 0.01	0.07 ± 0.01	0.04 ± 0.02	0.08 ± 0.01	0.07 ± 0.01
63	11.52	1366.8	Succinic acid, (2TMS)	0.11 ± 0.03	0.10 ± 0.02	0.09 ± 0.00	0.35 ± 0.23	0.16 ± 0.01	0.09 ± 0.02	0.11 ± 0.02	0.11 ± 0.02	0.13 ± 0.07	0.06 ± 0.00	0.28 ± 0.17	0.16 ± 0.04	0.08 ± 0.02	0.14 ± 0.03	0.10 ± 0.02
64	13.41	1496.1	Malonic acid, (2TMS)	0.08 ± 0.05	0.04 ± 0.00	0.05 ± 0.01	0.10 ± 0.02	0.19 ± 0.06	0.05 ± 0.01	0.05 ± 0.01	0.07 ± 0.03	0.31 ± 0.09	0.20 ± 0.05	0.13 ± 0.01	0.16 ± 0.08	0.12 ± 0.06	0.43 ± 0.24	0.13 ± 0.04
Total organic acids				4.57	4.74	5.13	5.96	6.34	2.92	4.53	5.17	6.05	4.01	5.11	4.30	3.72	4.87	5.35
Sugar alcohols																		
65	13.74	1520.3	Erythritol, (4TMS)	0.01 ± 0.00	0.01 ± 0.01	0.01 ± 0.00	0.01 ± 0.00	0.05 ± 0.06	0.00 ± 0.00	0.01 ± 0.00	0.01 ± 0.00	0.01 ± 0.00	0.01 ± 0.00	0.01 ± 0.01	0.01 ± 0.00	0.01 ± 0.00	0.02 ± 0.00	0.02 ± 0.00
66	14.43	1572.4	Threonic acid, (3TMS)	0.01 ± 0.00	0.01 ± 0.01	0.02 ± 0.01	0.11 ± 0.03	0.19 ± 0.11	0.04 ± 0.04	0.02 ± 0.02	0.03 ± 0.01	0.14 ± 0.07	0.15 ± 0.04	0.10 ± 0.02	0.23 ± 0.11	0.06 ± 0.04	0.20 ± 0.05	0.23 ± 0.08
67	15.35	1641.2	Xylonic acid, 1,5-lactone, (3TMS)	0.22 ± 0.00	0.10 ± 0.01	0.10 ± 0.01	0.12 ± 0.02	0.16 ± 0.00	0.47 ± 0.02	0.43 ± 0.01	0.41 ± 0.19	0.26 ± 0.01	0.39 ± 0.01	0.37 ± 0.03	0.18 ± 0.02	0.20 ± 0.02	0.17 ± 0.02	0.19 ± 0.02
68	15.85	1677.7	Arabino-Hexonic acid, 3-deoxy-*O*-(3TMS)lactone	0.98 ± 0.83	0.02 ± 0.00	0.02 ± 0.00	0.03 ± 0.01	0.02 ± 0.01	0.01 ± 0.01	0.05 ± 0.06	0.02 ± 0.00	0.04 ± 0.01	0.02 ± 0.00	0.02 ± 0.00	0.05 ± 0.01	0.04 ± 0.02	0.06 ± 0.01	0.05 ± 0.03
69	16.14	1713.3	Xylitol, (5TMS)	0.00 ± 0.00	0.00 ± 0.00	0.00 ± 0.00	0.01 ± 0.00	0.01 ± 0.00	0.00 ± 0.00	0.00 ± 0.00	0.00 ± 0.00	0.01 ± 0.00	0.00 ± 0.00	0.01 ± 0.00	0.01 ± 0.00	0.01 ± 0.00	0.01 ± 0.01	0.01 ± 0.00
70	17.07	1791.6	Fucitol, (5TMS)	0.20 ± 0.07	0.09 ± 0.01	0.09 ± 0.01	0.15 ± 0.01	0.26 ± 0.06	0.12 ± 0.04	0.07 ± 0.01	0.27 ± 0.01	0.22 ± 0.09	0.10 ± 0.03	0.14 ± 0.05	0.21 ± 0.02	0.13 ± 0.02	0.39 ± 0.20	0.42 ± 0.05
71	17.72	1853.1	Pinitol, (5TMS)	0.50 ± 0.36	0.04 ± 0.01	0.60 ± 0.22	1.05 ± 0.44	2.27 ± 2.21	0.38 ± 0.37	0.32 ± 0.13	0.27 ± 0.16	0.32 ± 0.08	0.17 ± 0.03	1.70 ± 1.25	5.36 ± 2.79	0.05 ± 0.00	5.13 ± 2.21	3.73 ± 0.02
72	17.85	1866.6	1,5-Anhydro-d-sorbitol, (4TMS)	0.01 ± 0.00	0.01 ± 0.01	0.01 ± 0.00	0.02 ± 0.01	0.02 ± 0.01	0.01 ± 0.00	0.01 ± 0.00	0.02 ± 0.00	0.01 ± 0.01	0.00 ± 0.00	0.01 ± 0.01	0.04 ± 0.01	0.01 ± 0.01	0.03 ± 0.01	0.04 ± 0.00
73	18.26	1893	Mannonic acid, 1,5-lactone, 4 (TMS)	0.08 ± 0.09	0.01 ± 0.00	0.02 ± 0.01	0.09 ± 0.04	0.13 ± 0.11	0.03 ± 0.03	0.03 ± 0.02	0.08 ± 0.05	0.05 ± 0.01	0.03 ± 0.01	0.89 ± 0.84	0.17 ± 0.11	0.04 ± 0.03	0.20 ± 0.13	0.10 ± 0.05
74	18.78	1952	Mannitol, (6TMS)	0.02 ± 0.01	0.02 ± 0.01	0.01 ± 0.00	0.04 ± 0.00	0.86 ± 1.41	0.02 ± 0.02	0.05 ± 0.03	0.04 ± 0.01	0.03 ± 0.00	0.01 ± 0.01	0.14 ± 0.15	0.03 ± 0.01	0.01 ± 0.00	0.04 ± 0.01	0.04 ± 0.01
75	18.87	1959.7	Sorbitol, (6TMS)	0.44 ± 0.64	0.04 ± 0.06	0.04 ± 0.01	0.17 ± 0.05	0.31 ± 0.29	0.04 ± 0.04	0.04 ± 0.04	0.06 ± 0.02	0.10 ± 0.01	0.02 ± 0.00	2.16 ± 2.98	0.33 ± 0.29	0.04 ± 0.03	0.29 ± 0.12	0.13 ± 0.13
76	19.54	2025.7	Gluconic acid, (6TMS)	0.24 ± 0.24	0.09 ± 0.01	0.10 ± 0.01	0.25 ± 0.12	0.25 ± 0.22	0.08 ± 0.00	0.12 ± 0.05	0.12 ± 0.03	0.10 ± 0.06	0.10 ± 0.01	0.74 ± 0.95	0.19 ± 0.13	0.10 ± 0.03	1.85 ± 2.94	0.16 ± 0.09
77	20.08	2087.3	Myo-Inositol, (5TMS)	0.01 ± 0.00	0.01 ± 0.00	0.01 ± 0.00	0.01 ± 0.00	0.01 ± 0.01	0.01 ± 0.00	0.01 ± 0.00	0.03 ± 0.02	0.01 ± 0.00	0.02 ± 0.01	0.03 ± 0.00	0.01 ± 0.01	0.01 ± 0.01	0.27 ± 0.45	0.01 ± 0.00
78	20.41	2114.2	Myo-Inositol, (6TMS)	0.29 ± 0.20	0.03 ± 0.02	0.03 ± 0.02	0.33 ± 0.08	0.36 ± 0.19	0.11 ± 0.12	0.09 ± 0.05	0.27 ± 0.26	0.50 ± 0.04	0.16 ± 0.01	0.68 ± 0.30	0.17 ± 0.05	0.36 ± 0.19	0.28 ± 0.10	0.49 ± 0.21
Total sugar alcohols				3.02	0.47	1.07	2.39	4.90	1.32	1.26	1.62	1.81	1.19	7.01	6.98	1.08	8.94	5.60
Sugars																		
79	17.99	1878	Glucose, (5TMS)	0.00 ± 0.00	0.00 ± 0.00	0.00 ± 0.00	0.01 ± 0.00	0.01 ± 0.00	0.00 ± 0.00	0.00 ± 0.00	0.00 ± 0.00	0.01 ± 0.00	0.00 ± 0.00	0.01 ± 0.00	0.01 ± 0.00	0.00 ± 0.00	0.01 ± 0.00	0.01 ± 0.00
80	18.36	1916.9	Mannopyranoside, methyl (4TMS)	0.03 ± 0.00	0.02 ± 0.01	0.02 ± 0.01	0.13 ± 0.01	0.07 ± 0.03	0.02 ± 0.02	0.03 ± 0.02	0.13 ± 0.06	0.04 ± 0.01	0.03 ± 0.00	0.04 ± 0.01	0.03 ± 0.00	0.07 ± 0.02	0.04 ± 0.00	0.06 ± 0.01
81	18.47	1923	Glucose, (5TMS)	0.03 ± 0.04	0.01 ± 0.00	0.01 ± 0.00	0.02 ± 0.00	0.01 ± 0.00	0.01 ± 0.01	0.01 ± 0.00	0.03 ± 0.02	0.02 ± 0.01	0.01 ± 0.00	0.03 ± 0.02	0.02 ± 0.00	0.03 ± 0.01	0.02 ± 0.01	0.02 ± 0.00
82	19.04	1976.2	d-Allofuranose, (5TMS)	0.02 ± 0.01	0.01 ± 0.00	0.02 ± 0.01	0.03 ± 0.01	0.05 ± 0.04	0.02 ± 0.02	0.05 ± 0.03	0.02 ± 0.00	0.03 ± 0.00	0.01 ± 0.00	0.10 ± 0.07	0.66 ± 0.24	1.84 ± 0.59	0.86 ± 0.72	1.07 ± 0.28
83	19.25	1996.7	d-Glucose, (5TMS)	0.03 ± 0.01	0.02 ± 0.01	0.03 ± 0.01	0.19 ± 0.02	0.09 ± 0.04	0.03 ± 0.02	0.04 ± 0.02	0.15 ± 0.07	0.05 ± 0.02	0.03 ± 0.00	0.05 ± 0.02	0.05 ± 0.00	0.09 ± 0.04	0.06 ± 0.00	0.09 ± 0.01
84	19.36	2006.6	Galactofuranose, (5TMS)	0.01 ± 0.00	0.00 ± 0.00	0.00 ± 0.00	0.01 ± 0.00	0.01 ± 0.00	0.01 ± 0.01	0.00 ± 0.00	0.01 ± 0.00	0.01 ± 0.00	0.01 ± 0.00	0.02 ± 0.01	0.01 ± 0.00	0.01 ± 0.00	0.01 ± 0.01	0.01 ± 0.01
85	24.80	2624.2	Sucrose isomer	0.01 ± 0.00	0.00 ± 0.00	0.00 ± 0.00	0.09 ± 0.03	0.14 ± 0.10	0.09 ± 0.14	0.01 ± 0.00	0.05 ± 0.06	0.05 ± 0.00	0.01 ± 0.01	0.10 ± 0.05	0.15 ± 0.09	0.03 ± 0.02	0.17 ± 0.09	0.13 ± 0.03
86	25.15	2670.9	Trehalose, (8TMS)	0.00 ± 0.00	0.00 ± 0.00	0.00 ± 0.00	0.02 ± 0.01	0.03 ± 0.02	0.01 ± 0.01	0.01 ± 0.00	0.01 ± 0.01	0.02 ± 0.01	0.01 ± 0.00	0.02 ± 0.01	0.04 ± 0.02	0.01 ± 0.01	0.05 ± 0.02	0.04 ± 0.01
87	25.26	2684.7	Sucrose, (8TMS)	0.47 ± 0.25	0.04 ± 0.02	0.16 ± 0.10	16.29 ± 4.27	17.58 ± 10.68	4.61 ± 6.73	0.57 ± 0.76	1.85 ± 2.30	3.80 ± 1.26	1.11 ± 0.30	13.25 ± 5.85	19.08 ± 9.22	4.64 ± 2.79	18.85 ± 6.27	14.70 ± 1.78
88	25.42	2685.4	d-Trehalose, (8TMS)	0.07 ± 0.04	0.03 ± 0.00	0.03 ± 0.00	1.36 ± 0.82	0.69 ± 0.17	0.31 ± 0.47	0.04 ± 0.01	0.04 ± 0.01	0.05 ± 0.01	0.04 ± 0.01	0.06 ± 0.01	0.07 ± 0.02	0.06 ± 0.02	0.09 ± 0.03	0.08 ± 0.01
89	25.85	2759.5	d-Glucopyranose, 4[TMS)-beta-d-galactopyranosyl (4TMS)	0.01 ± 0.00	0.00 ± 0.00	0.01 ± 0.00	0.02 ± 0.00	0.05 ± 0.02	0.01 ± 0.02	0.01 ± 0.01	0.03 ± 0.01	0.02 ± 0.01	0.02 ± 0.00	0.01 ± 0.00	0.02 ± 0.01	0.01 ± 0.00	0.05 ± 0.07	0.01 ± 0.00
90	26.23	2804.8	β-Gentiobiose, (8TMS)	0.05 ± 0.05	0.01 ± 0.00	0.32 ± 0.34	3.82 ± 1.65	6.34 ± 5.63	1.47 ± 2.13	0.35 ± 0.23	0.39 ± 0.54	0.57 ± 0.15	0.94 ± 0.20	0.55 ± 0.23	0.33 ± 0.22	0.02 ± 0.01	0.40 ± 0.11	0.31 ± 0.03
91	31.60		Unknown disaccharide	0.45 ± 0.40	0.01 ± 0.00	0.66 ± 0.81	4.54 ± 2.80	2.54 ± 1.09	3.12 ± 4.01	0.71 ± 1.07	0.31 ± 0.30	2.15 ± 0.08	1.86 ± 0.27	2.66 ± 0.70	4.92 ± 0.66	1.68 ± 0.51	10.02 ± 2.73	8.28 ± 1.36
Total sugars				1.17	0.15	1.27	26.55	27.62	9.71	1.84	3.02	6.82	4.07	16.90	25.38	8.49	30.63	24.80
Terpenes/steroids																		
92	6.08	1032	Limonene	0.19 ± 0.14	0.16 ± 0.11	0.04 ± 0.00	0.12 ± 0.13	0.23 ± 0.16	0.20 ± 0.02	0.11 ± 0.10	0.27 ± 0.02	0.15 ± 0.12	0.06 ± 0.02	0.24 ± 0.18	0.14 ± 0.17	0.12 ± 0.11	0.18 ± 0.11	0.32 ± 0.06
93	6.14	1035.6	Cineole (Eucalyptol)	0.07 ± 0.06	0.07 ± 0.05	0.01 ± 0.00	0.05 ± 0.06	0.09 ± 0.07	0.07 ± 0.01	0.04 ± 0.05	0.10 ± 0.01	0.06 ± 0.05	0.01 ± 0.01	0.08 ± 0.06	0.04 ± 0.05	0.04 ± 0.05	0.07 ± 0.05	0.13 ± 0.03
94	26.34	2815.9	Squalene	0.14 ± 0.04	0.08 ± 0.03	0.09 ± 0.03	0.26 ± 0.04	0.16 ± 0.03	0.41 ± 0.35	0.30 ± 0.23	0.82 ± 0.98	1.63 ± 1.20	0.55 ± 0.19	1.17 ± 0.45	0.31 ± 0.21	0.25 ± 0.05	0.51 ± 0.31	0.27 ± 0.23
95	27.63	2935.7	Tocopherol-γ-Tms-derivative	0.01 ± 0.00	0.01 ± 0.00	0.01 ± 0.00	0.01 ± 0.00	0.01 ± 0.00	0.02 ± 0.00	0.02 ± 0.00	0.01 ± 0.00	0.01 ± 0.01	0.02 ± 0.00	0.03 ± 0.00	0.03 ± 0.00	0.01 ± 0.00	0.12 ± 0.16	0.01 ± 0.00
96	28.64	3034.3	Stigmastan-3,5-diene	0.03 ± 0.00	0.02 ± 0.00	0.02 ± 0.00	0.03 ± 0.00	0.03 ± 0.00	0.06 ± 0.01	0.07 ± 0.01	0.04 ± 0.00	0.04 ± 0.00	0.06 ± 0.00	0.03 ± 0.03	0.03 ± 0.00	0.02 ± 0.01	0.53 ± 0.85	0.04 ± 0.00
97	30.81	3263.1	β-Sitosterol TMS	0.12 ± 0.01	0.06 ± 0.00	0.08 ± 0.01	0.17 ± 0.05	0.30 ± 0.10	0.14 ± 0.05	0.15 ± 0.01	0.09 ± 0.00	0.07 ± 0.04	0.11 ± 0.01	0.09 ± 0.06	0.19 ± 0.04	0.22 ± 0.07	0.69 ± 0.61	0.38 ± 0.03
98	31.12	3297.4	β-Amyrin TMS	0.01 ± 0.00	0.01 ± 0.01	0.01 ± 0.01	0.06 ± 0.03	0.02 ± 0.01	0.05 ± 0.03	0.02 ± 0.00	0.06 ± 0.01	0.61 ± 0.38	0.17 ± 0.01	0.15 ± 0.06	0.01 ± 0.01	0.01 ± 0.00	0.02 ± 0.01	0.04 ± 0.01
Total terpenes/steroids				0.57	0.40	0.26	0.68	0.84	0.95	0.70	1.39	2.57	0.97	1.78	0.76	0.67	2.12	1.18

Seeds were analyzed via GC–MS, n = 3 and concentration is expressed as mg/g. For codes explanation, refer to Table 1.

#### Fatty acids and acyl esters

Fatty acids and acyl esters predominated examined seeds as typical storage organs detected at 6.2 to 64.2 mg/g except for *Trifolium* TP and TI in which sugar levels were more abundant (26.6 and 27.6 mg/g, respectively vs. 14.4 and 17.9 mg/g fatty acids and acyl esters, respectively). Lipids were represented mostly by saturated (SFA) as palmitic acid, monounsaturated (MUFA) viz*.* oleic acid, and polyunsaturated fatty acids (PUFA) viz*.* linoleic and α-linolenic acids. *Ononis* OS, OA, OR, *Trifolium* TM, and *Medicago* MX encompassed the highest levels of fatty acids and acyl esters ranging from 24.5 to 64.2 mg/g, posing them as candidates for future use in biofuel industry^23^, in contrast to the majority of legumes encompassing low fat^24^. SFA ranged from 5.4 mg/g in *Melilotus* MO to 30.1 mg/g in OS represented by mainly myristic (peak 30), palmitic (peak 33), stearic (peak 38), arachidic (peak 39), behenic (peak 42) and lignoceric acids (peak 43). While unsaturated fatty acids were detected at highest levels in *Ononis* OA and OS (19.6 and 34.1 mg/g, respectively) versus lowest in *Melilotus* MO and MS (0.8 and 1.3 mg/g, respectively), comprising oleic (peak 35), linoleic (peak 36) and α-linolenic acids (peak 37).

### Saturated fatty acids (SFA)

The examined seeds encompassed palmitic and stearic acids as the major SFA, others detected at much smaller levels included arachidic, myristic, and behenic acids, in accordance with previous reports^16,25,26^. Palmitic acid (C16:0) (peak 33) was the major detected SFA in the examined seeds reaching highest level in OS and OA (10.5 and 9.9 mg/g, respectively), and lowest in *Melilotus* MO and MS (1.9 and 2.3, respectively). Likewise, monopalmitin (peak 41) was present in *Ononis* OS at its highest concentration (4 mg/g) followed by *Melilotus* MA, *Ononis* OA, and *Medicago* MDO (1–1.6 mg/g), while it ranged from 0.5 to 0.9 mg/g in other seeds. Although palmitic acid has negative effects on chronic adult ailments, it remains an essential element in the membrane, transport, and secretory lipids^27^. Its average daily intake is at ca. 20–30 g accounting for 8–10 energy%.

Also, high levels of stearic acid (C18:0) (peak 38) were detected in examined seeds ranging from 2.1 in *Melilotus* MO to 9.2 in OS. The average daily intake of stearic acid was estimated at 8.1 and 5.4 g, accounting for ca. 91% of the total fat for mean and women, respectively^28^. Besides imparting the required physical characteristics of solid fat^29^, stearic acid exerts a hypocholesterolemic potential similar to that of oleic acid^30,31^. Both palmitic and stearic acid dietary supplementation increase milk production in cows^32,33^, suggesting that examined seeds especially *Ononis* OS and OA represent a healthy fat source for humans and as fodder.

Arachidic acid was also detected in *Ononis* i.e., OS at a higher concentration (2.1mg/g) compared to the other seed *species* (0.08–0.4 mg/g). Likewise, behenic acid and lignoceric acid were detected at exclusively higher levels in OS compared to other seeds (1.7 and 0.9 mg/g) *vs.* (0.05–0.4 mg/g and 0.04–0.15 mg/g, respectively), suggesting that they could be used as markers to distinguish OS from other *Ononis* species. While myristic acid was higher in *Trifolium* TM, TA, *Medicago* MDS, and ML (1.1–1.3 mg/g) vs. (0.4–0.8 mg/g) in other seed species.

### Unsaturated fatty acids (MUFA and PUFA)

The examined seeds were enriched in oleic, linoleic, and α-linolenic acids with variable amounts, in line with previous reports^16,25,26^. Oleic acid (peak 35), monounsaturated ω-9 fatty acid, was the major distinguished MUFA in all seeds ranging from 2.4 to 27.3 mg/g except *Melilotus* MO and MS (0.6 and 0.8 mg/g, respectively) detected at highest level in *Ononis* OS 27.3 mg/g followed by OA, *Trifolium* TM, and *Ononis* OR (9.9–12.5 mg/g). Such abundance of oleic acid in the later seed species pose them as healthy functional foods, owing to its several health benefits e.g., antioxidant^34^, anti-inflammatory^35^, hepatoprotective^35^, anticancer effects^36^, besides its potential to lower serum LDL cholesterol^37^.

ω-3 and ω-6, polyunsaturated fatty acids, are not biosynthesized by humans and must be introduced into the diet^38^. Linoleic acid (peak 36), ω-6 fatty acid was detected at highest level in *Ononis* OA, OR, and OS (3.4–4.1 mg/g), while its level ranged from 1 to 2 mg/g in most other seeds. Such enrichment of *Ononis* species in linoleic acid accentuates their antioxidant^39^ and anti-inflammatory properties^40^.

Likewise, α-linolenic acid (peak 37), major ω-3 fatty acid was detected at highlevel in *Ononis* species, in addition to *Medicago* MX, MDO, and *Melilotus* MA compared to other seeds (1.2–3.4 vs. 0.1–0.9 mg/g). Both linoleic and α-linolenic acids exhibit antidiabetic^41,42^ and antihypercholesterolemic effects^37^.

A diet with a lower ω-6/ω-3 ratio is suggested to reduce the risk of several chronic ailments e.g., a ratio of 2–3 suppressed inflammation in rheumatoid arthritis patients, also a ratio of 2.5 decreased cell proliferation colorectal cancer patients^43^. The ω-6/ω-3 ratio in the investigated seeds ranged from 0.35 to 2.1 suggesting a good ω-6/ω-3 ratio. It was found lowest in *Melilotus* MA, MO, and *Medicago* MDS (0.35–0.42) and higher in *Trifolium* TP, TI, TM, *Ononis* OR, and OA (1.1–2.1).

Similarly, oleic/linoleic acid ratio is important as a higher ratio increases the resistance of LDL to oxidation and consequently decreases atherosclerosis, in addition, it increases the seeds’ shelf life^44^. The oleic/linoleic acid ratio in examined seeds ranged from 2.8 in OR to 14.2 in *Melilotus* MO. It was higher in *Medicago* ML, *Trifolium* TA, *Medicago* MDS, and *Melilotus* MO reaching (11.3, 12.1, 12.6, and 14.2, respectively). Such richness in oleic, linoleic, and α-linolenic acids with good ω-6/ω-3 and oleic/linoleic acid ratios of *Ononis* species (OR, OS and OA) and further *Medicago* (MX) suggest that they could be added to the diet to regulate serum cholesterol levels.

#### Sugars and sugar alcohols

Sugar level is important for seeds’ nutritional value and taste affecting their palatability. Sugars (mono- and disaccharides) predominated *Trifolium* TP and TI (26.6 and 27.6, respectively) amounting for the second abundant class in *Trifolium* TM, *Medicago* MX, and *Ononis* species at levels ranging from 9.7 to 30.6 mg/g, while they were remarkably low in *Melilotus* and *Trifolium* TA (0.2–3 mg/g) as revealed in the bar chart represented in Fig. 2**.**

Two *Trifolium* species (TP and TI) and three *Ononis* species (OS, OR, and OA) displayed the highest sugar content(24.8–30.6 mg/g), followed by *Medicago* (MX) at 16.9 mg/g, while other seed species displayed lower levels ranging from 1.2 to 9.7 mg/g. *Melilotus* displayed the lowest sugar levels (0.15–1.3 mg/g), especially MO (0.15mg/g).

Disaccharides were the most abundant sugar subclass detected in seeds albeit with some variations viz*.* sucrose, gentiobiose, and trehalose. Sucrose (peak 87) predominated in most of the examined seeds but at different levels in agreement with previous reports describing sucrose as the major legume seed sugar^45^. It was present at higher levels in *Ononis* (OR, OS, OA), *Trifolium* (TP and TI), and *Medicago* (MX) ranging from 13.3 to 19.1 mg/g, while *Medicago* species showed the lowest levels (0.04–0.5 mg/g) and suggestive for their lower palatabilty. Sucrose, the disaccharide of glucose and fructose, consumption in moderate amounts potentiates insulin release through fructose occurrence together with a stimulatory amount of glucose. Consequently, such seed intake will not raise post-prandial glucose level^46^. Gentiobiose (peak 90), the bitter disaccharide^47^, was enriched in three *Trifolium* species viz*.* TI, TP, and TM compared to others (1.5–6.4 vs. 0.01–0.9 mg/g). While trehalose (peak 88) was remarkably higher in *Trifolium* TP relative to other seed species (1.4 *vs.* 0.03–0.7 mg/g).

Compared to disaccharides, low levels of monosaccharides were detected except allofuranose (peak 82) which was detected in *Ononis* at relatively high levels (0.7–1.8 mg/g).

14 Sugar alcohols were identified (peak 65–78) in the examined seeds ranging from 0.5 to 8.9 mg/g. *Ononis* OS, OR, OA, *Medicago* MX,and *Trifolium* TI were the highest in sugar alcohols (4.9–8.9 mg/g) while *Melilotus* MO was the lowest (0.5 mg/g). Sugar alcohols are sweeteners of low glycemic index that add fewer calories to the diet and are endorsed for diabetic patients, in addition to their prebiotic effects^48^.

Pinitol (peak 71) was exceptionally high in 3 *Ononis* species viz*.* OR, OS, and OA (4–5) slightly higher from that reported in other seed legumes from previous studies e.g., soybean, lentil and chickpea (3.48, 1.97 and 1.95 mg/g, respectively)^49^. Also, pinitol level was higher in *Trifolium* TI and TP (2.3 and 1.1 mg/g, respectively) compared to the *Trifolium* TA and TM (0.3 and 0.4 mg/g, respectively) suggesting that pinitol could be used as a marker to distinguish between *Ononis*, as well as *Trifolium* species aside from its several health benefits e.g., antidiabetic, anti-inflammatory, antioxidant, and cardioprotective effects^50^. Future studies should target the selective removal of interfering low molecular weight carbohydrates such as mono and disaccharides to overcome their interference with the legume’s inositols and consequently their bioactivity as functional foods for diabetic patients and also to decrease their calorie content. Such fractionation can be applied using yeast treatment^49^ or ion exchange resins^51^ .

Sorbitol (peak 75) was exclusively high in *Medicago* MX (2.2 mg/g) compared to other examined seeds (0.02–0.4 mg/g). Contrary to other legumes such as soy beans, neither phytic acid nor the raffinose family oligosaccharides were distinguished in any of the examined seeds. Raffinose oligosaccharides are considered anti-nutrients as they are responsible for the flatulence effect of legume seeds^52^. Likewise, myoinositol, the precursor of phytic acid, was detected at trace amounts in the examined seeds with higher concentrations in *Ononis* OA and *Medicago* MX (0.5 and 0.7 mg/g, respectively). Such absence of raffinose and phytic acid should be further confirmed by examining seeds from different origins and using other techniques. Legumes' nutritional value is hampered by the substantial number of antinutrients´ they encompass. In most legumes, oligosaccharides of the raffinose family, led mostly by raffinose, prevail. Since humans lack α-galactosidase, oligosaccharides are a primary cause of flatulence due to their indigestion accompanied by uncomfortable symptoms i.e., flatulence, nausea, cramps, diarrhea, and abdominal pain due to anaerobic fermentation by the cecal and colonic bacteria. On the other hand, phytic acid forms an insoluble combination with minerals decreasing their bioavailability’s as in case of Fe, Zn, Mg, Ca, Cu, and Mn ions. In order to decrease antinutrients and improve bioavailability of nutritional components in the food system, a number of conventional food processing procedures are typically employed including soaking, germination, fermentation, and cooking can be used^52^. Phytic acid could be separated using ion exchange chromatography and estimated as described by^53^ or^54^, whereas raffinose could be estimated using HPLC or TLC as described in^53^ for QC procedure in food products.

#### Organic acids/alcohols

Organic acids stimulate pancreatic enzymes’ secretion, induce digestion and absorption of many metabolites, moreover they have a strong bactericidal effect^55^ and act as preservatives in food^56^.

17 Organic acids were identified in the seeds under investigation (peak 48–64) detected at levels ranging from 2.9 to 6.3 mg/g mainly represented by lactic acid and γ-hydroxybutyric acid. *Trifolium* TI, *Medicago* MDO, and *Trifolium* TP displayed the highest organic acids level at *ca.* 6 mg/g, accounting for their slightly sour taste. Lactic acid (peak 50) was the major organic acid in all seeds detected at highest level of *ca.* 3 mg/g in *Melilotus* MS, *Trifolium* TP, TI, and *Medicago* MDO. γ-Hydroxybutyric acid (peak 58) was the second major organic acid in the seeds detected at (0.7–1.4 mg/g) with lower levels in *Trifolium* TM, TA, and *Medicago* ML (0.7–0.8 mg/g) and higher in *Trifolium* TP, TI, and OS (1.3–1.4 mg/g). It is noteworthy that oxalic acid, the health-hazardous organic acid, was not present in examined seeds^57^.

*Ononis* seeds viz., OS, OA, and OR showed the highest alcohol level (8–9.9 mg/g) compared to other seeds (2.1–6.5 mg/g). Glycerol (peak 3), the sweet triol^58^, was the major alcohol in examined seeds, especially in OS, OA, and OR (7.4–9 mg/g) exceeding the other seed species (1.4–5.7 mg/g).

#### Amino acids/nitrogenous compounds

Free amino acids in examined seeds ranged from 5.2 to 12 mg/g. *Ononis* species (OA, OS, and OR), *Medicago* species (MX and MDO), and *Trifolium* species (TP and TI) showed the highest free amino acid level (ca. 9–12 mg/g) posing them as potential nutritive sources, yet their crude protein content should be further investigated. While the other *Trifolium* species (TA and TM) and *Melilotus* (MO and MS) were the least enriched (ca. 5.2–5.7 mg/g).

Essential, non-essential, and conditionally essential free amino acids were all detected in the examined seeds. The identified essential amino acids comprised valine, leucine, isoleucine, phenyl alanine, threonine, cysteine, while the conditionally essential amino acids included proline and tyrosine, and the nonessential amino acids encompassed alanine, serine, glycine, aspartic acid, pyroglutamic acid, and glutamic acid. l-Threonine (peak 15) was the major identified amino acid in all seeds (3.4–5.2 mg/g), followed by glycine (peak 17) (1.23–3.25 mg/g) with higher levels in *Ononis* OA and *Trifolium* TI (5.2 and 5 mg/g for threonine and 3.3 and 2.9 for glycine, respectively). Whereas, pyroglutamic acid (peak 21), the memory-enhancing amino acid^59^, was abundant in most seeds with higher levels in *Trifolium* TI, TP, *Medicago* MDO, MX, *Ononis* OR, OS, and OA ranging from 1 to 2 mg/g. On the other hand, serine (peak 13) was distinguished in *Medicago* seeds at relatively higher concentrations than others (0.3–0.8 mg/g *vs.* 0.02–0.06 mg/g).

Interestingly, cysteine level (peak 19) in both *Trifolium* TP and *Medicago* MX (1.87 and 1.11 mg/g, respectively) was higher than in other seed species (ca. 0.01–0.2 mg/g). As reported in many seed legumes, the examined species except *Trifolium* TP and *Medicago* MX were low in sulfur-containing amino acids e.g., cysteine^4^, pointing out that they may not be sufficient protein sources but should be supplemented with other balanced protein sources^60^. Moreover, lysine, histidine, tryptophan, and methionine were not detected contrary to previous reports in *Medicago*, *Melilotus*, *Trifolium*, and *Ononis*^4^. Hence, additional crude protein profiling with appropriate protein extraction and analysis techniques is recommended to verify their exact protein content. Different techniques could be utilized to assign the entire amino acid composition e.g., GC-FID and GC-IRMS^61^ and ion-exchange HPLC^62^.

Nitrogenous compounds were detected in the examined seed species at low levels ranging from 0.14 to 1.9 mg/g. They were mainly represented by nicotinic acid (peak 44) in *Trifolium* TI, TP, and OA (0.7–1.5 mg/g). Nicotinic acid has positive effects in cases of dyslipidemia as it greatly increases the plasma high-density lipoprotein (HDL) cholesterol levels. It is worth mentioning that none of the antinutrient biogenic amines were detected in the examined seeds *e.g.,* cadaverine, putrescine, tyramine, and tryptamine, indicating their good storage and safety^52^.

#### Steroids and tocopherols

Unlike other seed legumes in which β-sitosterol is the most abundant phytosterol *e.g.,* peas and lentils (1.91 and 1.23 mg/g)^63^, the examined seeds showed trace amounts except for *Ononis* OS (0.7 mg/g). Likewise, they showed small amounts of tocopherol (peak 95, 0.01–0.1 mg/g) and squalene (peak 94, 0.08–0.6 mg/g), detected only at high level in *Medicago* MDS, MDO, and MX (0.8–1.6 mg/g). This implies that in the examined seeds, except *Medicago* MDS, MDO, and MX, do not present rich sources of these antioxidants compared to other seeds^64^. Profiling using LC–MS can though better provide insight on these seeds antioxidant potential with regards to phenolics content. Few terpenes such as limonene and cineole were detected at trace levels and likely to contribute for flavour in MDS and OA (0.3 and 0.1 mg/g, respectively)X.

### GC–MS-based multivariate data analysis (MVDA) for the primary metabolites of seeds of *Medicago*, *Melilotus*, *Ononis*, and* Trifolium* species

#### Unsupervised multivariate data analysis PCA and HCA of whole dataset

GC–MS based MVDA analysis tools were further employed to assess metabolites variations (differences) among the seeds of *Medicago*, *Melilotus*, *Ononis*, and *Trifolium* species. The unsupervised HCA and PCA analysis, in addition to the supervised OPLS-DA were employed to assist in species (accessions) distinction and markers identification.

**Figure 3:**
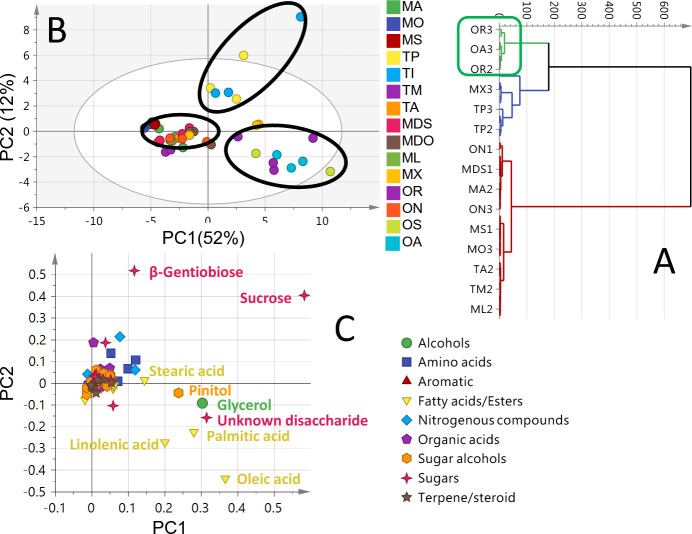
GC–MS based HCA and PCA of primary metabolites from all seeds’ specimens. (**A**) HCA plot. (**B**) Score plot of PC1 vs. PC2 scores. (**C**) Loading plot for PC1 & PC2 contributing metabolites and their assignments. The metabolome clusters are located at the distinct positions in two-dimensional space described by two vectors of principal component 1 (PC1) = 52% and PC2 = 12%.

The unsupervised HCA and PCA (Fig. 3) was established for discrimination between seeds of *Medicago*, *Melilotus*, *Ononis*, and *Trifolium* accessions. The HCA (Fig. 3A) portrayed one main cluster of the *Ononis* accessions, which could be ascribed to *Ononis* richness in fatty acids (29.8–64.2 mg/g), sugars (24.8–30.6 mg/g), sugar alcohols (5.6–8.9 mg/g) and free amino acids content (8.7–12 mg/g) as revealed from GC–MS analysis (Table 2). It should be noted that HCA failed to discriminate between all other seeds of *Medicago*, *Melilotus*, and *Trifolium*, as their independent biological replicates were dispersed and overlapped. The generated PCA model (Fig. 3B) accounted for 64% of the total variance, with PC1 and PC2 to account for 52% and 12%, respectively. The PCA model showed partial segregation of *T. incarnatu* (TI) and *T. pannonic* (TP) in one cluster in the upper right side and another cluster in the lower right side for *O. repens* (OR), *O. arvensis* (OA), and *O. spinosa* (OS). However, an obvious overlap of the independent biological replicates was observed among *O. natrix* (ON), *T. montanu* (TM), *T. arvense* (TA), and *M. albus* (MA). The unsupervised PCA loading plot (Fig. 3C) revealed that alcohols (glycerol), fatty acids (linolenic, oleic, palmitic and stearic acids), sugars (β-gentiobiose, sucrose and unknown disaccharide), and sugar alcohols (pinitol) were the markers responsible for such segregation. Hence, PCA model failed to provide clustering of individual seed replicates, except for *T. incarnatu* (TI) and *T. pannonic* (TP). Therefore, supervised OPLS-DA analysis was further adopted to minimize variance among replicates for each species to achieve better (species) separation.

#### Unsupervised multivariate data analysis PCA and HCA of each genotype separately

Unsupervised PCA models were further constructed for the accessions within the same genotype (genus) each modelled separately to assess the variability and similarities between the accessions and for better identification of markers within each genotype.

**Figure 4:**
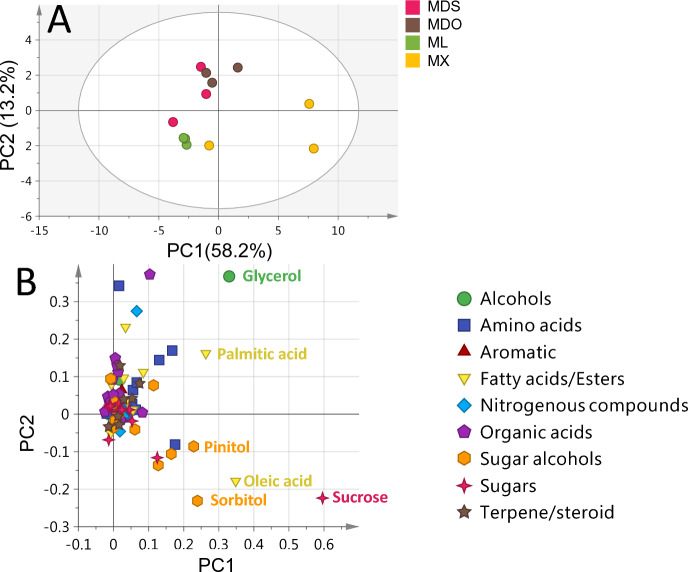
GC–MS based HCA and PCA of primary metabolites from all *Medicago* seeds accessions (**A**) Score plot of PC1 vs. PC2 scores. (**B**) Loading plot for PC1 & PC2 contributing metabolites and their assignments. The metabolome clusters are located at the distinct positions in two-dimensional space described by two vectors of principal component 1 (PC1) = 58.2% and PC2 = 13.2%.

The unsupervised PCA score plot for *Medicago* accessions (Fig. 4A) showed a total variance at (71.4%), with PC1 (58.2%) against PC2 (13.2%), however failed to discriminate between *Medicago* accessions. Two replicates from *M. xvaria* (MX) accessions were clustered together, while the other replicate was clustered and overlapped with other *Medicago* accessions. The PCA loading plot (Fig. 4B) indicated that alcohols (glycerol), fatty acids (palmitic and oleic acids), sugars (sucrose), and sugar alcohols (pinintol and sorbitol) contributed to such segregation.

**Figure 5:**
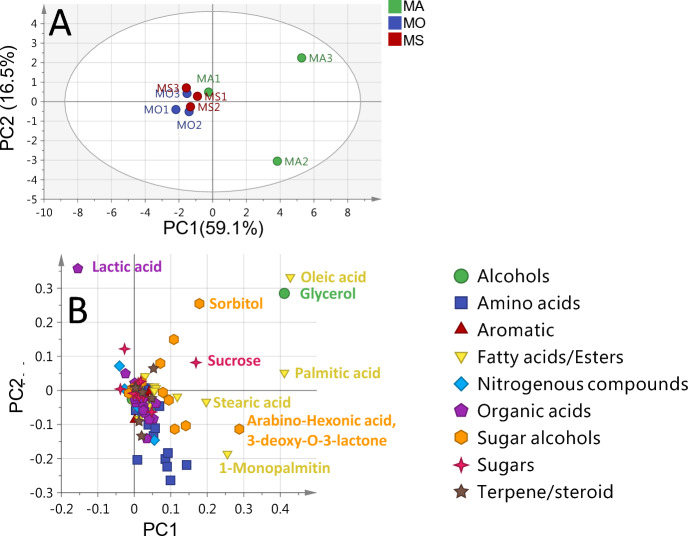
GC–MS based PCA of primary metabolites from *Melilotus* seeds accessions. (**A**) Score plot of PC1 vs. PC2 scores. (**B**) Loading plot for PC1 & PC2 contributing metabolites and their assignments. The metabolome clusters are located at the distinct positions in two-dimensional space described by two vectors of principal component 1 (PC1) = 59.1% and PC2 = 16.5%.

The unsupervised PCA score plot for *Melilotus* accessions model showed a total variance at (75.6%), with PC1 (59.1%) versus PC2 (16.5%). The PCA score plot (Fig. 5A) showed segregation of one of the three replicates of *M. albus* (MA) at the upper right side, while other replicate of *M. albus* (MA) was segregated far at the right lower side. Although the other *M*elilotus accessions were clustered and overlapped together. The PCA loading plot (Fig. 5B) indicated that alcohols (glycerol), fatty acids (oleic and palmitic acids), sugars (sucrose), and sugar alcohols (sorbitol) were potential markers for the segregation of *M. albus* (MA3), while segregation of *M. albus* (MA2) was assigned to its richness in fatty acids/esters (i.e., stearic acid and 1-monopalmitin) and the sugar acid (arabino-hexanoic acid, 3-deoxy-O-lactone).

**Figure 6:**
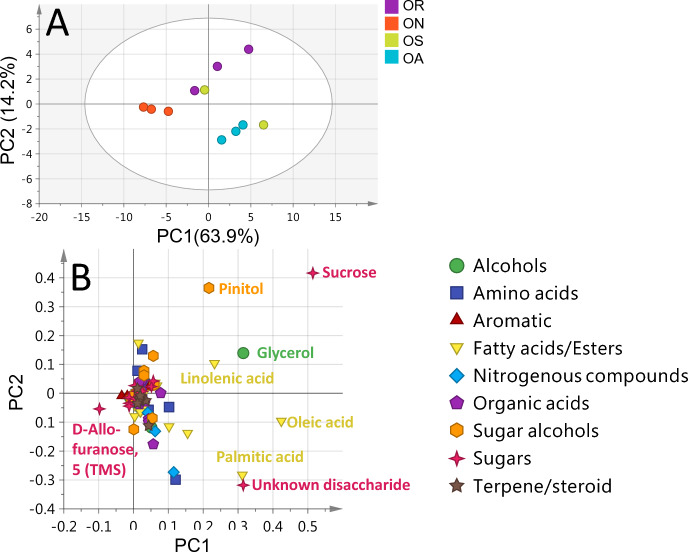
GC–MS based HCA and PCA of primary metabolites from all *Ononis* seeds accessions. (**A**) Score plot of PC1 vs. PC2 scores. (**B**) Loading plot for PC1 & PC2 contributing metabolites and their assignments. The metabolome clusters are located at the distinct positions in two-dimensional space described by two vectors of principal component 1 (PC1) = 63.9% and PC2 = 14.2%.

The unsupervised PCA score plot for *Ononis* accessions (Fig. 6A) showed a total variance at (78.1%). The PCA score plot showed clear separation of *O. natrix* (ON) accessions, while the independent biological replicates of *O. spinosa* (OS), *O. repens* (OR) and *O. arvensis* (OA) accessions were overlapped and dispersed. The PCA loading plot (Fig. 6B) indicated that the sugar D-allofuranose contributed for *O. natrix* (ON) separation, while glycerol, linolenic acid, sucrose, and pinitol more erniched in *O. repens* (OR) accessions.

The unsupervised PCA score plot for *Trifolium* accessions (S. Fig. 1) portrayed two clusters, one for both *T. pannonic* (TP) and *T. incarnatu* (TI) at the right side, whereas other cluster was for *T. montanu* (TM) and *T. arvense* (TA) at the left side. The PCA score plot showed a total variance at (77.6%) and in agreement with HCA result (S. Fig. 1a). The PCA loading plot (S. Fig. 1c) demonstrated that both *T. montanu* (TM) and *T.arvense* (TA) segregation was attributed fortheir richness in myristic acid. In contrast, *T. pannonic* (TP) and *T. incarnatu* (TI) segregation was accounted for their richness in alcohol (glycerol), fatty acid (oleic acid) and sugars (β-gentiobiose, sucrose, and pinitol).

#### Supervised multivariate data analysis OPLS-DA

Supervised OPLS-DA (S. Fig. 2A) was performed in an attempt to differentiate between the seeds independent replicates and to further identify metabolite markers, albeit constructed model prediction power was relatively weak (negative value). Though, the OPLS-DA inner class relationship (S. Fig. 2B) revealed overlap of *O. spinosa* (OS) and *O. arvensis* (OA) independent replicates and their distant segregation.

Another supervised OPLS-DA (S. Fig. 3) was likewise employed to identify the markers responsible for the segregation (clustering) of the *Ononis species* as concluded from the unsupervised HCA and PCA (Fig. 3), and the supervised OPLS-DA inner class relationship (S. Fig. 2B). The supervised OPLS-DA was constructed in which *Ononis* species were modelled in one class against *Medicago*, *Melilotus*, and *Trifolium* species in the other class. The developed model (S. Fig. 3) showed a better samples separation, R^2^ (88%) and Q^2^ (78%), indicating high prediction power. The OPLS-DA score plot confirmed segregation of *Ononis* species from all other seeds’ accessions. The OPLS-DA score plot (S. Fig. 3B) revealed that alcohols (glycerol), fatty acids (linolenic, palmitic, and oleic acids), sugars (d-allofuranose, sucrose, unknown disaccharide), and sugar alcohols (pinitol) are the main discriminators of *Ononis* species, confirming the notable differences in their GC–MS based metabolites profiles (Table 2). The developed OPLS-DA model was validated using permutation test, confirming its statistically significant, as p-value being lower than 0.05 (S. Fig. 4).

### Metabolites enrichment analysis

Metabolites enrichment analysis of the monitored metabolites using GC–MS was employed to reveal for the most differential pathways in each seed genus viz. *Medicago*, *Melilotus*, *Ononis* and *Trifolium* (S. Fig. 5), using the (Functional analysis) module of MetaboAnalyst 5.0.

The major mapped pathways with greatest number of differentially expressed genes (DEGs) in the seeds of *Medicago* genus included alpha linolenic acid/ linoleic acid metabolism, amino sugar metabolism, β-alanine metabolism, starch and glucose metabolism, lysine degradation, arachidonic acid metabolism, galactose metabolism, and oxidation of branched chain fatty acids pathways (S. Fig. 5A).

With regards to seeds of *Melilotus* genus, starch and sucrose metabolism, b-alanine metabolism, glycine/serine metabolism, oxidation of branched chain fatty acids, inositol/inositol phosphate metabolism, and phosphatidylinositol phosphate metabolic pathways were the main presented pathways (S. Fig. 5B).

Additionally, top mapped pathways in *Ononis* genus belonged to fatty acids metabolism, oxidation of branched chain fatty acids, α-linolenic acid/linoleic acid metabolism, β-oxidation of long chain fatty acids, glycine/serine metabolism pathways (S. Fig. 5C).

The *Trifolium* genus were enriched with galactose metabolism, starch and sucrose metabolism, fatty acids biosynthesis, beta oxidation of very long chain fatty acids, inositol metabolism, inositol phosphate metabolism, and phosphatidylinositol phosphate metabolism, steroid biosynthesis pathways versus *Medicago*, *Melilotus*, and *Ononis* groups (S. Fig. 5D).

## Conclusion

**Figure 7:**
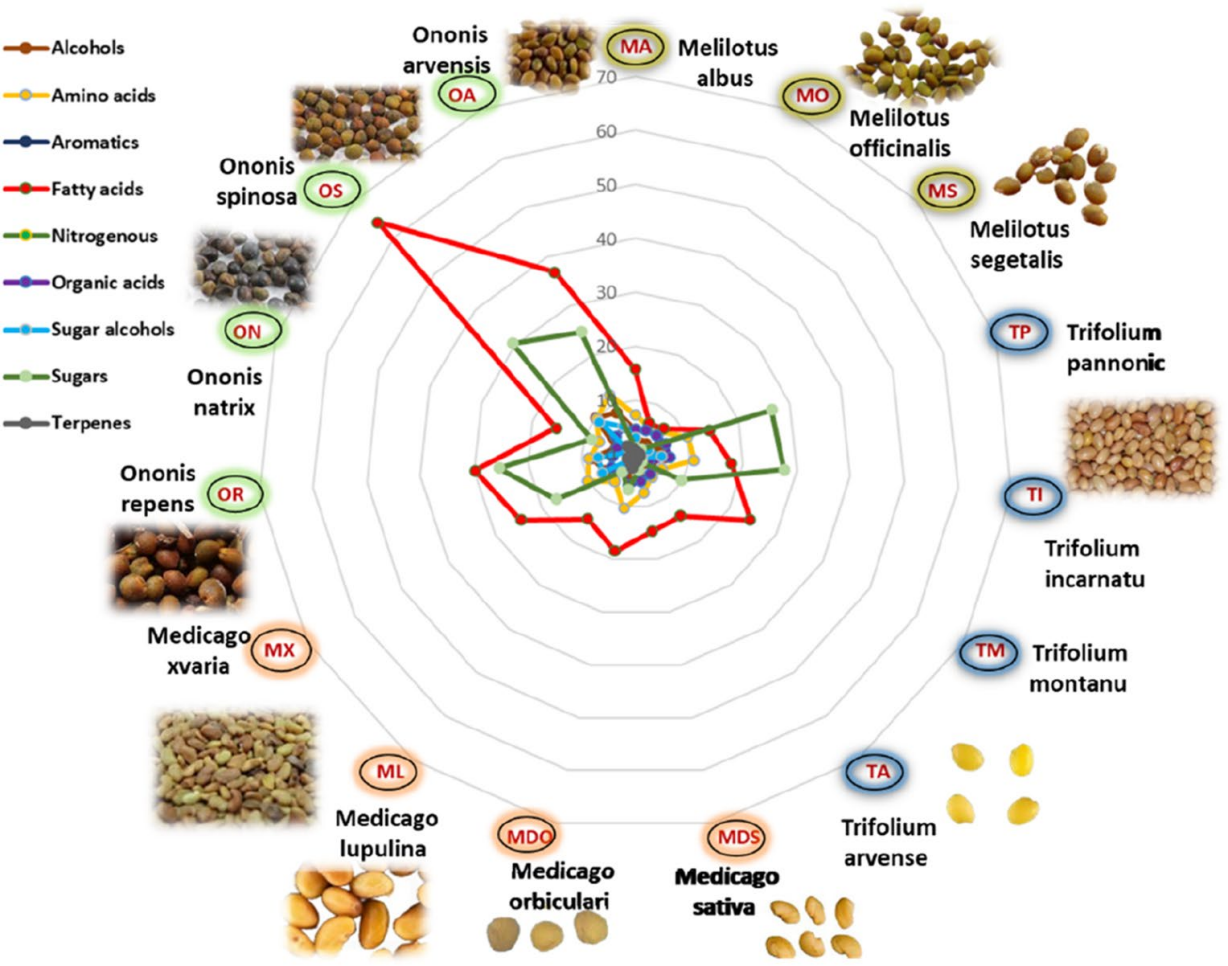
A radar plot illustrating the concentration of major metabolite classes in *Melilotus, Trifolium, Medicago,* and *Ononis* seed species expressed as mg/g.

Our results revealed that among examined seed legumes viz*. Melilotus, Medicago, Ononis*, and *Trifolium*, *Ononis* seeds (OR, OS and OA) were almost the most abundant in fatty acids (29.8–64.2 mg/g), sugars (24.8–30.6 mg/g), sugar alcohols (5.6–8.9 mg/g) and free amino acids content (8.7–12 mg/g), while less enriched in organic acids (4.3–5.4 mg/g), as displayed in the radar plot (Fig. 7), suggesting that they are nutritionally valuable and palatable both for human and as fodder. In contrast, *Melilotus* species (MO and MS) were not enriched in fatty acids (6.2–7 mg/g), sugars (0.2–1.3 mg/g), sugar alcohols (0.5–1.1mg/g), and free amino acids (5.7–6.1 mg/g), suggesting that they are not treasured as potential nutrients neither for human nor as fodder.

OS was the richest in fatty acids followed by OA and OR. Likewise, OS was the most abundant in sugars followed by TI, TP, OR, and OA (Fig. 7).

Interestingly, OS displayed the highest fatty acids (64.2 mg/g), sugars (30.6 mg/g) sugar alcohols (8.9 mg/g), alcohols (9.9 mg/g) and moderate free amino acids content (9.6 mg/g) and organic acid (4.9 mg/g) compared to all other seeds, proposing its nutritional value and palatability.

Lacking many essential free amino acids, the examined seeds may not considered as a sufficient protein source but further studies should be conducted to unveil the total protein content both as free aminoacids protein. However, *T. pannonic* and *M. xvaria* are considered the best with relatively high free amino acids as total and essential amino acids viz*.* cysteine and threonine. Further crude protein profiling using LCMS platform shall provide better insight of these seeds protein content.

The fatty acids profile of *Ononis* species (OR, OS and OA) and *Medicago* (MX) revealed for their richness in oleic, linoleic, and α-linolenic acids with good ω-6/ω-3 and oleic/linoleic acid ratios and suggest for their potential inclusion in diet to regulate serum cholesterol levels and prevent atherosclerosis. Asides from such fatty acids profile, *Ononis* richness in sugar alcohols such as pinitol or sorbitol pose for their low calorie content. Future studies should now focus on exploring secondary metabolites and their biochemical activities in these seeds.

The aim of the present study was to estimate free amino acid content and other low molecular weight primary metabolites as free sugars and their contribution in the nutritional value of the examined seeds using GC/MS. GC/MS has been previously used in other studies to detect primary metabolites’ content in plants exemplified by free amino acids, sugars and fatty acids^1,65^. Future studies should now focus on determining crude protein levels using appropriate protein extraction techniques to verify their exact total protein composition.

Chemometric tools have succeeded in the identification of *Ononis* metabolites’ markers belonging to various classes *i.e.*, (alcohol) glycerol, sugars (D-allofuranose,), and sugar alcohols (pinitol). The sugar D-Allofuranose was ascribed as discriminator marker for *O. natrix* (ON) accessions. Additionally, in *Trifolium* species, the segregation of *T. montanu* (TM) and *T. arvense* (TA) was attributed for their richness in myristic acid. The differentiation between *Medicago*, *Melilotus*, and *Trifolium* genera was not achieved, also the discrimination between species of the same genus was not attained suggestive for the use of stronger taxonomic markers for their classification targeting their secondary metabolome using LC/MS. Although we have targeted only accessions in legume seeds, same approach can be applied in the future for the exploration of factors affecting legume seeds metabolites profiling, including seasonal variations, cultivation, and storage conditions. Also, our study can provide new interesting details for future taxonomical studies especially if targeting larger genotypes.

### Supplementary Information

Supplementary Figures.

## Data Availability

The datasets used and analysed during the current study would be available from the corresponding author on reasonable request.

## Abbreviations

GC: Gas chromatography
HCA: Hierarchical cluster analysis
MS: Mass spectrometry
MUFA: Monounsaturated fatty acids
MVDA: Multivariate data analysis
OPLS-DA: Orthogonal projections to latent structures discriminant analysis
PCA: Principal component analysis
PUFA: Polyunsaturated fatty acids
SFA: Saturated fatty acids

## References

[1] Ibrahim N, Taleb M, Heiss AG, Kropf M, Farag MA (2021). GC-MS based metabolites profiling of nutrients and anti-nutrients in 10 Lathyrus seed genotypes: A prospect for phyto-equivalency and chemotaxonomy. Food Biosci..

[2] Kuo Y-H, Rozan P, Lambein F, Frias J, Vidal-Valverde C (2004). Effects of different germination conditions on the contents of free protein and non-protein amino acids of commercial legumes. Food Chem..

[3] Farag MA, El-Din MGS, Selim MA-F, Owis AI, Abouzid SF (2021). Mass spectrometry-based metabolites profiling of nutrients and anti-nutrients in major legume sprouts. Food Biosci..

[4] Elamine Y, Alaiz M, Girón-Calle J, Guiné RP, Vioque J (2022). Nutritional characteristics of the seed protein in 23 mediterranean legumes. Agronomy.

[5] Ragab NA, El Sawi SA, Aboutabl EA, El Halawany AM, Marzouk MM (2022). A comparative review on phytochemical constituents and biological effects of *Melilotus*
*indicus* (L.) All. and *Melilotus*
*messanensis* (L.) All., (Fabaceae): Evidence for chemosystematic analysis. Egypt. J. Chem..

[6] Sabudak, T. & Guler, N. *Trifolium* L.—A review on its phytochemical and pharmacological profile. *Phytother. Res. Int. J. Devoted Pharmacol. Toxicol. Eval. Nat. Prod. Deriv.* **23**, 439–446 (2009). 10.1002/ptr.2709 19107737

[7] Mielmann, A. The utilisation of lucerne (*Medicago sativa*): A review. *Br. Food J.* (2013).

[8] Bora KS, Sharma A (2011). Phytochemical and pharmacological potential of *Medicago*
*sativa*: A review. Pharmaceut. Biol..

[9] Tepe HD (2019). Qualitative analysis of alfalfa seed methanol extract by GC–MS and determination of antioxidant properties. Celal Bayar Univ. J. Sci..

[10] Mölgaard J, Von Schenck H, Olsson AG (1987). Alfalfa seeds lower low density lipoprotein cholesterol and apolipoprotein B concentrations in patients with type II hyperlipoproteinemia. Atherosclerosis.

[11] Brinker FJ (2001). Herb Contraindications & Drug Interactions.

[12] Jasicka-Misiak I, Makowicz E, Stanek N (2017). Polish yellow sweet clover (*Melilotus*
*officinalis* L.) honey, chromatographic fingerprints, and chemical markers. Molecules.

[13] Sisay MA, Mammo W, Yaya EE (2021). Phytochemical studies of *Melilotus*
*officinalis*. Bull. Chem. Soc. Ethiopia.

[14] Al-Qudah MA (2014). Antioxidant activity and chemical composition of essential oils from Jordanian *Ononis*
*natrix* L. and *Ononis*
*sicula* Guss. J. Biol. Active Prod. Nat..

[15] Gampe N, Nagy E, Kursinszki L, Béni S (2021). Quantitative determination of isoflavonoids in *Ononis* species by UPLC-UV-DAD. Phytochem. Anal..

[16] Chebli B, Hassani LMI, Hmamouchi M (2001). Acides gras et polyphénols des graines d'*Ononis*
*natrix* L. (Fabaceae) de la région d'Agadir, Maroc. Acta Bot. Gallica.

[17] Burda S, Oleszek W (2001). Antioxidant and antiradical activities of flavonoids. J. Agric. Food Chem..

[18] Simpson MG (2019). Plant Systematics.

[19] Farag MA, Afifi SM, Rasheed DM, Khattab AR (2021). Revealing compositional attributes of *Glossostemon*
*bruguieri* Desf. root geographic origin and roasting impact via chemometric modeling of SPME–GC–MS and NMR metabolite profiles. J. Food Compos. Anal..

[20] Farag MA, Maamoun AA, Ehrlich A, Fahmy S, Wesjohann LA (2017). Assessment of sensory metabolites distribution in 3 cactus *Opuntia*
*ficus**-indica* fruit cultivars using UV fingerprinting and GC/MS profiling techniques. Lwt.

[21] Farag MA, Ramadan NS, Shorbagi M, Farag N, Gad HA (2022). Profiling of primary metabolites and volatiles in apricot (*Prunus*
*armeniaca* L.) seed kernels and fruits in the context of its different cultivars and soil type as analyzed using chemometric tools. Foods.

[22] Saied DB, Ramadan NS, El-Sayed MM, Farag MA (2023). Effect of maturity stage on cereal and leguminous seeds’ metabolome as analyzed using gas chromatography mass-spectrometry (GC–MS) and chemometric tools. Metabolites.

[23] Goodrum JW, Geller DP (2005). Influence of fatty acid methyl esters from hydroxylated vegetable oils on diesel fuel lubricity. Bioresour. Technol..

[24] Maphosa Y, Jideani VA (2017). The role of legumes in human nutrition. Funct. Food-Improve Health Through Adequate Food.

[25] Bakoglu A, Kiliç Ö, Kökten K (2016). Seed fatty acid composition of some *Medicago* L. and *Melilotus* L. (Fabaceae) taxa from Turkey. Anal. Chem. Lett..

[26] Saruhan V (2017). Fatty acid compositions of the seeds of some *Trifolium* species. Chem. Nat. Compds..

[27] Innis SM (2016). Palmitic acid in early human development. Crit. Rev. Food Sci. Nutr..

[28] USDA, A. R. S. *Data Tables: Intakes of 19 Individual Fatty Acids: Results from the 1994–1996 Continuing Survey of Food Intakes by Individuals*. (2005).

[29] Kris-Etherton PM (2005). Dietary stearic acid and risk of cardiovascular disease: Intake, sources, digestion, and absorption. Lipids.

[30] Meng H (2019). Comparison of diets enriched in stearic, oleic, and palmitic acids on inflammation, immune response, cardiometabolic risk factors, and fecal bile acid concentrations in mildly hypercholesterolemic postmenopausal women—Randomized crossover trial. Am. J. Clin. Nutr..

[31] Crupkin M, Zambelli A (2008). Detrimental impact of trans fats on human health: Stearic acid-rich fats as possible substitutes. Comprehens. Rev. Food Sci. Food Saf..

[32] Piantoni P, Lock A, Allen M (2015). Milk production responses to dietary stearic acid vary by production level in dairy cattle. J. Dairy Sci..

[33] Piantoni P, Lock A, Allen M (2013). Palmitic acid increased yields of milk and milk fat and nutrient digestibility across production level of lactating cows. J. Dairy Sci..

[34] Cho K-H, Hong J-H, Lee K-T (2010). Monoacylglycerol (MAG)-oleic acid has stronger antioxidant, anti-atherosclerotic, and protein glycation inhibitory activities than MAG-palmitic acid. J. Med. Food.

[35] Gonçalves-de-Albuquerque CF, Silva AR, Burth P, Castro-Faria MV, Castro-Faria-Neto HC (2015). Acute respiratory distress syndrome: Role of oleic acid-triggered lung injury and inflammation. Mediat. Inflamm..

[36] Moon H-S, Batirel S, Mantzoros CS (2014). Alpha linolenic acid and oleic acid additively down-regulate malignant potential and positively cross-regulate AMPK/S6 axis in OE19 and OE33 esophageal cancer cells. Metabolism.

[37] Chan JK, Bruce VM, McDonald BE (1991). Dietary α-linolenic acid is as effective as oleic acid and linoleic acid in lowering blood cholesterol in normolipidemic men. Am. J. Clin. Nutr..

[38] Simopoulos AP (1999). Essential fatty acids in health and chronic disease. Am. J. Clin. Nutr..

[39] Fagali N, Catalá A (2008). Antioxidant activity of conjugated linoleic acid isomers, linoleic acid and its methyl ester determined by photoemission and DPPH techniques. Biophys. Chem..

[40] Knez Hrnčič M, Ivanovski M, Cör D, Knez Ž (2020). Chia seeds (*Salvia*
*hispanica* L.): An overview—phytochemical profile, isolation methods, and application. Molecules (Basel, Switzerland).

[41] Henderson G, Crofts C, Schofield G (2018). Linoleic acid and diabetes prevention. Lancet Diabetes Endocrinol..

[42] Suresh Y, Das U (2003). Long-chain polyunsaturated fatty acids and chemically induced diabetes mellitus: Effect of ω-3 fatty acids. Nutrition.

[43] Simopoulos AP (2002). The importance of the ratio of omega-6/omega-3 essential fatty acids. Biomed. Pharmacother..

[44] Chamberlin KD (2014). A comparison of methods used to determine the oleic/linoleic acid ratio in cultivated peanut (*Arachis*
*hypogaea* L.). Agric. Sci..

[45] Pua E-C (2010). Plant Developmental Biology-Biotechnological Perspectives.

[46] Kyriazis GA, Soundarapandian MM, Tyrberg B (2012). Sweet taste receptor signaling in beta cells mediates fructose-induced potentiation of glucose-stimulated insulin secretion. Proc. Natl. Acad. Sci..

[47] Sakurai T (2010). The human bitter taste receptor, hTAS2R16, discriminates slight differences in the configuration of disaccharides. Biochem. Biophys. Res. Commun..

[48] Grembecka M (2015). Sugar alcohols—Their role in the modern world of sweeteners: A review. Eur. Food Res. Technol..

[49] Ruiz-Aceituno L (2013). Optimisation of a biotechnological procedure for selective fractionation of bioactive inositols in edible legume extracts. J. Sci. Food Agric..

[50] Antonowski T (2019). Health-promoting properties of selected cyclitols for metabolic syndrome and diabetes. Nutrients.

[51] Camero, B. M. & Merino, C. S. (Google Patents, 2004).

[52] Sharma A (2021). A review on traditional technology and safety challenges with regard to antinutrients in legume foods. J. Food Sci. Technol..

[53] Zhawar VK, Kaur N, Gupta AK (2011). Phytic acid and raffinose series oligosaccharides metabolism in developing chickpea seeds. Physiol. Mol. Biol. Plants.

[54] Janardhanan K, Gurumoorthi P, Pugalenthi M (2003). Nutritional potential of five accessions of a South Indian tribal pulse, *Mucuna*
*pruriens* var* utilis* I. The effect of processing methods on the content of l-dopa, phytic acid, and oligosaccharides. Trop. Subtrop. Agroecosyst..

[55] Suiryanrayna MV, Ramana J (2015). A review of the effects of dietary organic acids fed to swine. J. Anim. Sci. Biotechnol..

[56] Theron MM, Lues JF (2007). Organic acids and meat preservation: A review. Food Rev. Int..

[57] Bsc SN, Bsc GS (1999). Oxalate content of foods and its effect on humans. Asia Pac. J. Clin. Nutr..

[58] Koseki T, Koganezawa M, Furuyama A, Isono K, Shimada I (2004). A specific receptor site for glycerol, a new sweet tastant for Drosophila: structure–taste relationship of glycerol in the labellar sugar receptor cell. Chem. Senses.

[59] Kumar A, Bachhawat AK (2012). Pyroglutamic acid: throwing light on a lightly studied metabolite. Curr. Sci..

[60] Tesseraud S, Coustard SM, Collin A, Seiliez I (2008). Role of sulfur amino acids in controlling nutrient metabolism and cell functions: implications for nutrition. Br. J. Nutr..

[61] Styring AK, Fraser RA, Bogaard A, Evershed RP (2014). Cereal grain, rachis and pulse seed amino acid δ15N values as indicators of plant nitrogen metabolism. Phytochemistry.

[62] Ravindran V, Abdollahi M, Bootwalla S (2014). Nutrient analysis, metabolizable energy, and digestible amino acids of soybean meals of different origins for broilers. Poultry Sci..

[63] Ryan E, Galvin K, O’Connor TP, Maguire AR, O’Brien NM (2007). Phytosterol, squalene, tocopherol content and fatty acid profile of selected seeds, grains, and legumes. Plant Foods Hum. Nutr..

[64] Kraujalis P, Venskutonis PR (2013). Supercritical carbon dioxide extraction of squalene and tocopherols from amaranth and assessment of extracts antioxidant activity. J. Supercrit. Fluids.

[65] Ramadan NS (2020). Nutrient and sensory metabolites profiling of *Averrhoa*
*carambola* L. (Starfruit) in the context of its origin and ripening stage by GC/MS and chemometric analysis. Molecules.

