# Supplementary material for: Comparative GC–MS based nutrients profiling of less explored legume seeds of Melilotus, Medicago, Trifolium, and Ononis analysed using chemometric tools
Source: Sci Rep. 2023 Oct 25;13:18221. doi: 10.1038/s41598-023-45453-0 (PMC10600120; doi:10.1038/s41598-023-45453-0)

**Comparative GC-MS based nutrients profiling of less explored legume seeds of *Melilotus, Medicago, Trifolium,* and *Ononis* analysed using chemometric tools**

**Supplementary figures**

**Suppl. Fig. 1:** GC-MS based HCA and PCA of primary metabolites from all *Trifolium* seeds accessions (A) HCA plot. (B) Score plot of PC1 vs. PC2 scores. (C) Loading plot for PC1 & PC2 contributing metabolites and their assignments. The metabolome clusters are located at the distinct positions in two-dimensional space described by two vectors of principal component 1 (PC1) = 56.5% and PC2 = 21.1%.

**Suppl. Fig. 2:** GC-MS based OPLS-DA score plot derived from all seeds’ specimens modelling (A). OPLS-DA inner class relationship of all seeds specimens (B).

**Suppl. Fig. 3:** GC-MS based OPLS-DA score plot derived from modelling of *Ononis* seeds’ species against *Medicago, Melilotus and Tripholium* seeds species (A). The respective S-plot (B) shows the covariance p[1] against the correlation p(cor)[1] of the variables of the discriminating component of the OPLS-DA model. Cut-off values of P<0.05 were used; selected variables are highlighted in the S-plot with identifications.

**Suppl. Fig. 4:** Validation of model, permutation p-value statistically significant p˂0.05

**Suppl. Fig. 5.** Metabolite enrichment analysis using Metaboanalyst (a) Medicago enrichment map; (b) Melilotus enrichment map; (c) Ononis enrichment map; (d) Trifolium enrichment map.

**
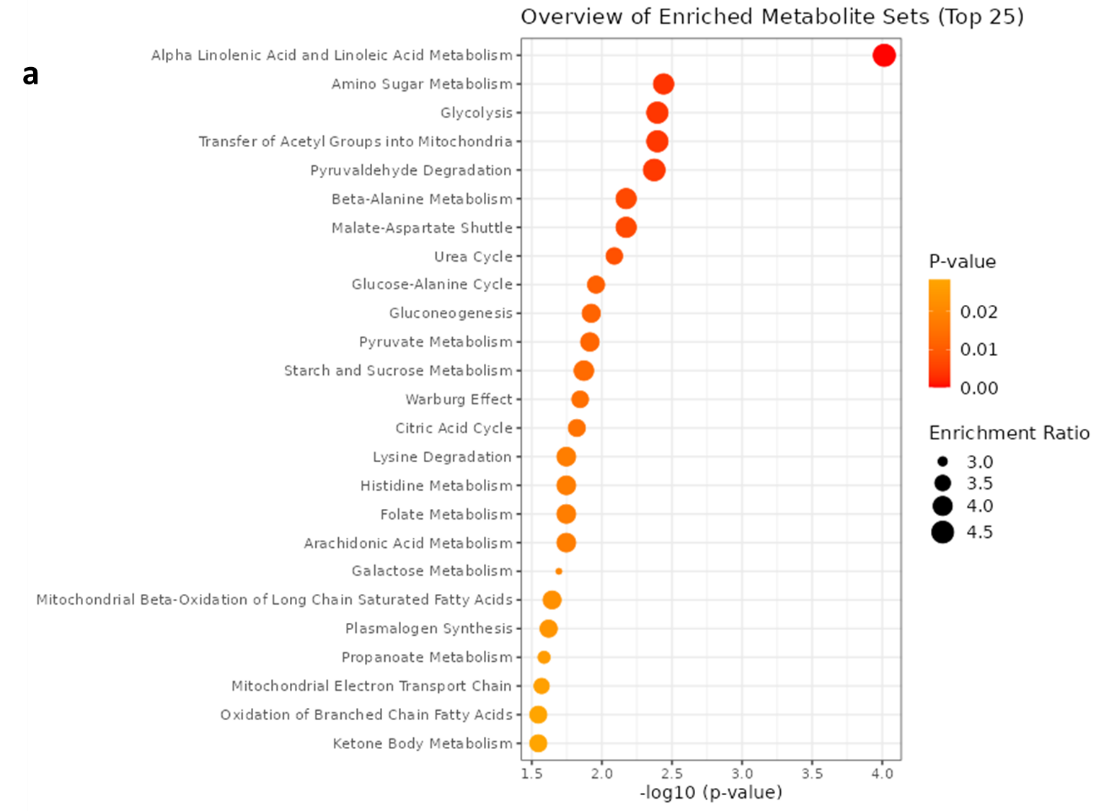
**

**
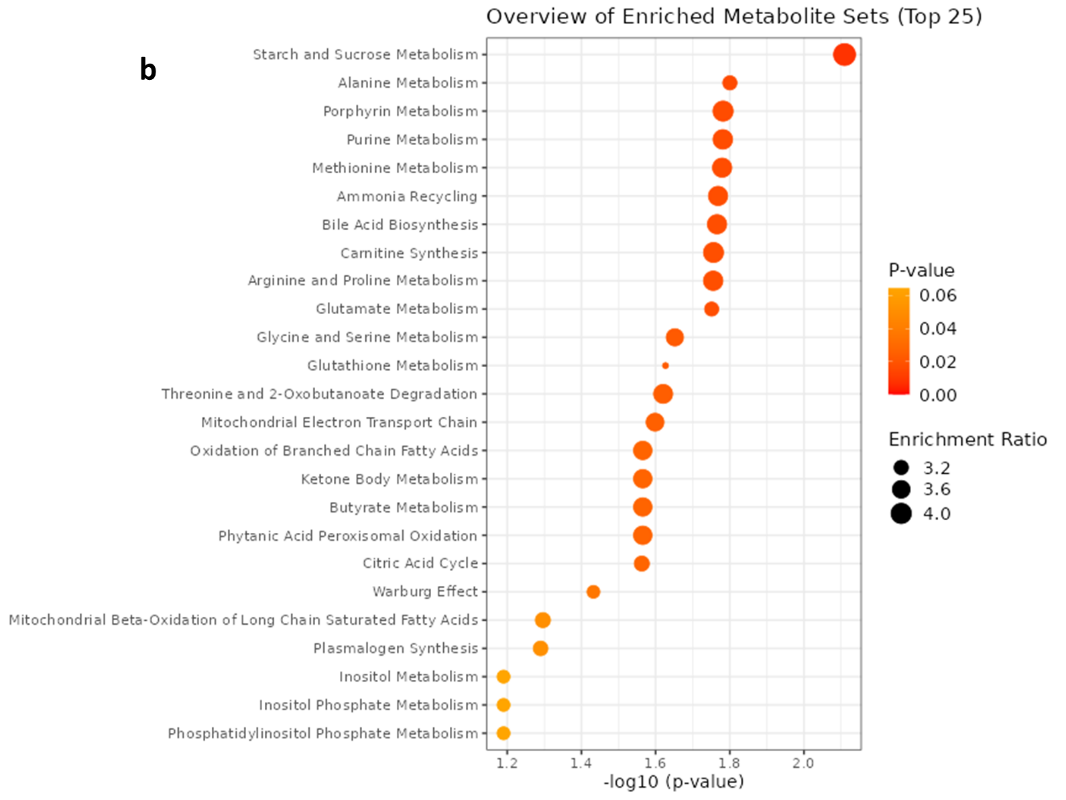
**


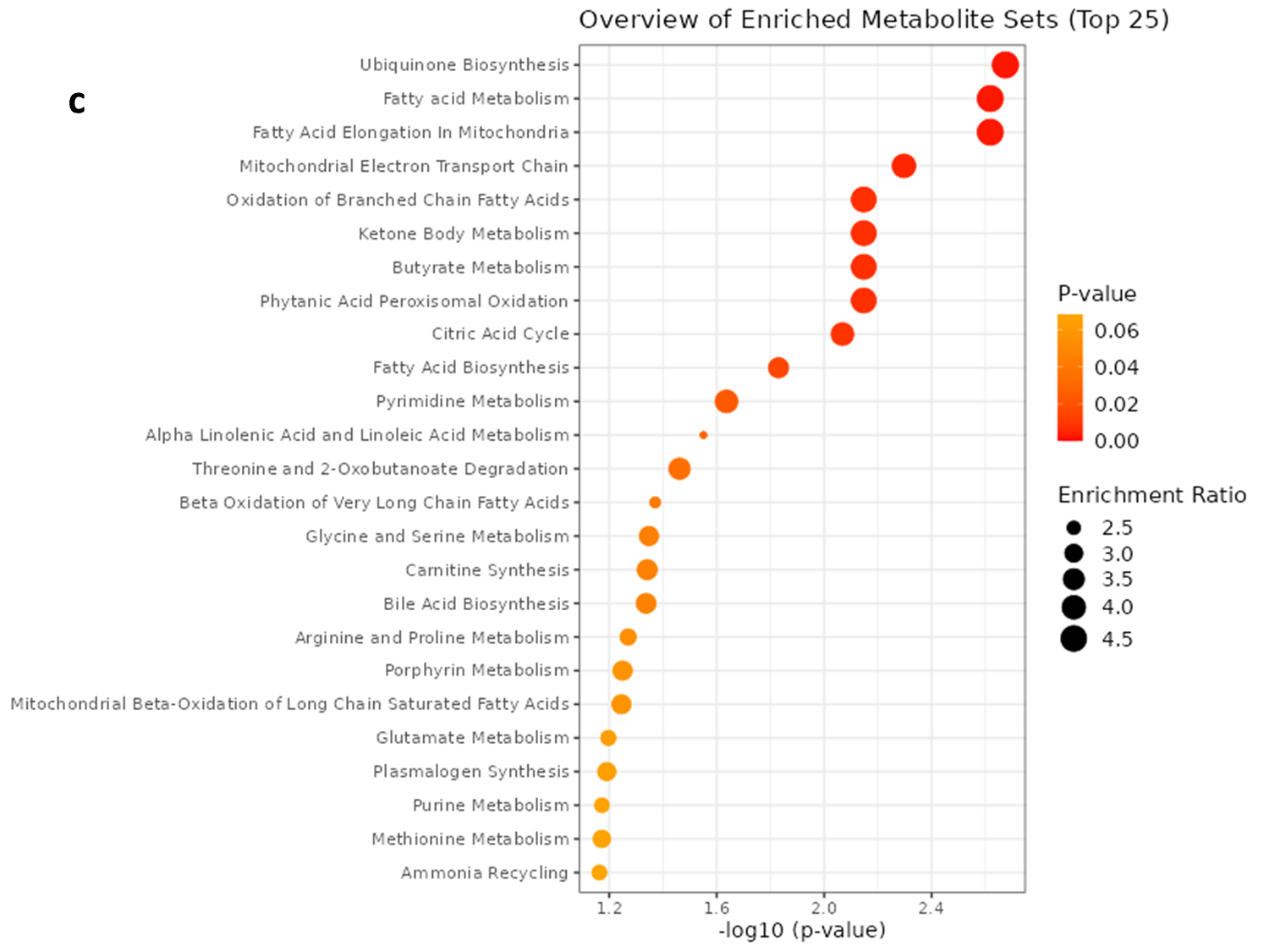


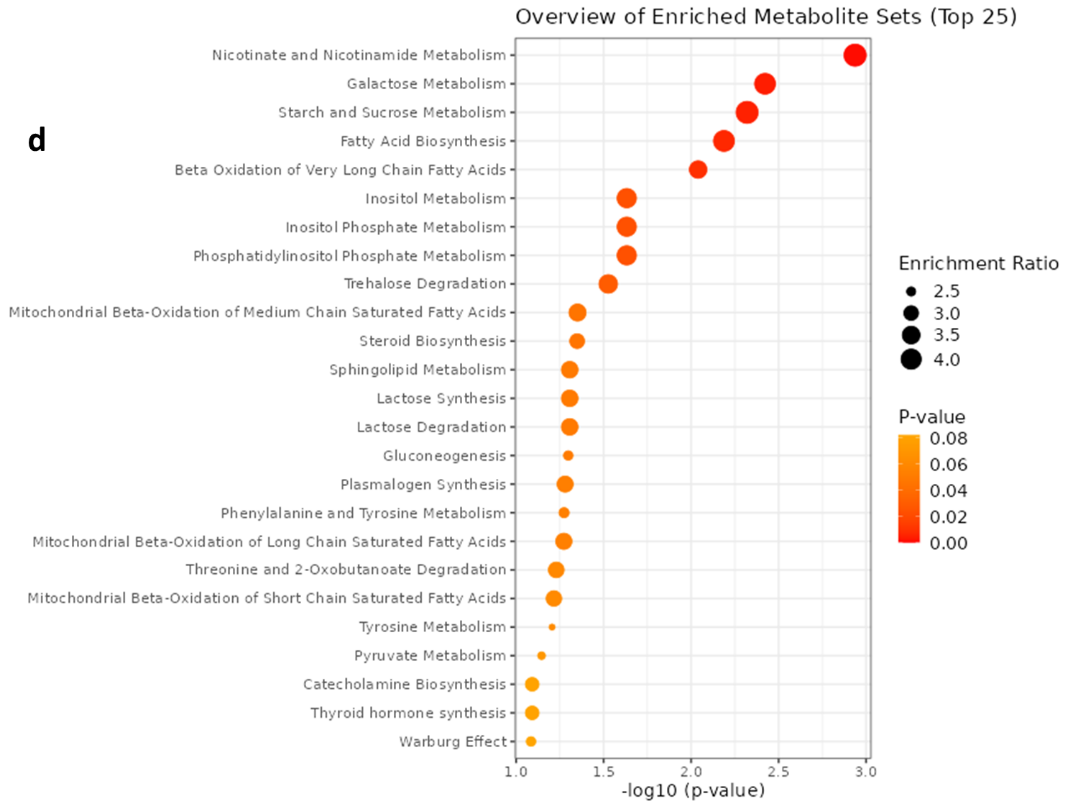

Supplement: Supplementary file 1 — Supplementary Figures. [file 41598_2023_45453_MOESM1_ESM.docx]
